# Newly validated touch experiences and attitudes questionnaire in German (TEAQ-G) is linked to social functioning, mental health, and hormonal stress regulation

**DOI:** 10.1038/s41598-025-20885-y

**Published:** 2025-10-09

**Authors:** E. Schneider, C. Raithel, D. Hopf, D. Scheele, P. D. Trotter, S. Franz, C. Aguilar-Raab, B. Ditzen, M. Eckstein

**Affiliations:** 1 Center for Psychosocial Medicine, Institute of Medical Psychology, Heidelberg University Hospital, Heidelberg, Germany; 2 Heidelberg University, Heidelberg, Germany; 3 German Center for Mental Health (DZPG), Partner Site Heidelberg/Mannheim/Ulm, Mannheim, Germany; 4 Department of Social Neuroscience, Faculty of Medicine, Ruhr University Bochum, Bochum, Germany; 5 Research Center One Health Ruhr of the University Alliance Ruhr, Ruhr University Bochum, Bochum, Germany; 6 Research Centre for Brain & Behaviour, School of Psychology, Liverpool John Moores University, Liverpool, UK; 7 Department of General Psychiatry, Center for Psychosocial Medicine, Heidelberg University Hospital, Heidelberg, Germany; 8 Clinical Psychology, Interaction- and Psychotherapy Research, School of Social Sciences, Institute for Compassionate Awareness and Interdependence Research and Practice, University of Mannheim, Mannheim, Germany

**Keywords:** Psychology, Human behaviour

## Abstract

Interpersonal affectionate touch plays a crucial role in social bonding, stress regulation, and psychological well-being. However, individual differences in past touch experiences and attitudes toward touch remain understudied. This study aimed to validate the German version of the touch experiences and attitudes questionnaire (TEAQ-G) and investigate how the subscales childhood touch experiences, current intimate touch, and attitudes toward touch relate to social relationships, mental health, emotional states, cortisol and oxytocin levels in everyday life. Data from 1,319 study participants suggest good psychometric properties of the TEAQ-G, confirming its reliability and validity as a tool for assessing touch experiences and attitudes. Regression analyses revealed that retrospectively reported more positive childhood touch and current intimate touch were associated with lower levels of attachment avoidance, stress, anxiety, depression, and loneliness, as well as higher resilience, family functioning, and relationship satisfaction. These findings were further supported by ecological momentary assessment data from 253 subjects (6 measures à two days, resulting in 3036 data points), associating positive childhood touch experiences with more favorable daily emotional states in adulthood. Specifically, individuals reported lower levels of stress, anxiety, loneliness, and burden related to the COVID-19 pandemic, along with increased happiness. Similarly, current intimate touch was linked to lower stress, pandemic-related burden, reduced loneliness, higher happiness, and moderately higher salivary oxytocin levels. Moreover, both childhood touch experiences and attitudes toward intimate touch significantly moderated the relationship between daily affectionate touch reports and individuals’ happiness, stress levels, pandemic-related burden, and cortisol concentrations. Our results emphasize the developmental importance of early touch experiences and highlight the lasting impact of both early and ongoing touch on attachment, social relationships, psychological well-being, and hormonal responses in everyday life.

**Supplementary Information:**

The online version contains supplementary material available at 10.1038/s41598-025-20885-y.

## Introduction

The sense of touch, including affectionate touch, is the first to develop during intrauterine life and plays a crucial role in shaping social relationships^1^. Interpersonal affectionate touch significantly influences human development, from early childhood through adulthood^1^. In early developmental stages, it contributes to self-regulation, as well as to socio-emotional and cognitive development^2^. Although parental touch typically decreases during adolescence, early experiences of affectionate tactile interactions have lasting effects on self-regulation and the distinction between self and other^2^. Furthermore, touch experiences during childhood and adolescence may shape adult attachment styles and influence how individuals engage with touch in their relationships^1^.

More specifically, previous research demonstrates that close and more frequent physical contact between mothers and their infants is essential for fostering a more secure attachment during childhood^3,4^ and is also associated with lower levels of attachment avoidance in adulthood^1^. Early caregiver tactile interactions also influence the development of brain regions that regulate the stress response, impacting the nervous, endocrine, and immune systems^5^. Additionally, these experiences may have long-term implications for mental health. Although research on this prospective connection remains limited, some studies indicate an association between retrospectively self-reported parental physical contact in childhood and the risk of developing depression in later adolescence and early adulthood^6,7^. Conversely, substantial evidence indicates that touch-based interventions — such as massage, gentle touch, or stroking — offer significant benefits for both physical and mental health. These interventions can help reduce depression, anxiety, and pain in both adults and children, as highlighted in a recent review and meta-analysis^8,9^.

One possible mechanism underlying these positive effects of touch is the activation of C-tactile (CT) afferent fibers, which play a crucial role in encoding the pleasantness of slow, gentle touch^10^. It has been demonstrated that CT afferent fibers respond to specific touch velocities and temperatures^11,10^. Remarkably, humans can recognize the rewarding value of CT-optimal caressing touch even when they are not personally experiencing it simply by observing others being touched^12^ or even imagining being touched^13^. This suggests that affectionate touch is inherently rewarding, which adults learn to recognize over time. Interestingly, this positive value can also be transferred to neutral social stimuli through associative learning that occurs during touch^14^.

Beyond its role in emotional bonding, affectionate touch also exerts direct stress-buffering effects. Several studies have reported decreases in heart rate, cortisol levels, and self-reported stress ratings following affectionate touch^15–17^. One proposed mechanism for this stress reduction involves the release of oxytocin, which inhibits the hypothalamic-pituitary-adrenal (HPA) axis, thereby reducing stress hormone secretion^5^.

Substantial research has highlighted the role of touch in social bonding, attachment, and stress regulation. For example, a recent study found that couples’ positive attitudes towards touch during pregnancy can predict the frequency and variety of affectionate and sexual behavior at three months postpartum^18^. Less is known about how individual differences in past and current touch experiences and attitudes shape psychological well-being and hormonal stress regulation in daily life. Previous studies have often focused on caregiver-infant interactions, the mechanistic activation of CT afferents, or the short-term effects of touch-based interventions^8^ leaving a gap in understanding the long-term impact of touch across the lifespan. Understanding not only the relationship between current touch experiences and health but also childhood touch experiences and health is important for potential future health applications.

To address this, we translated and validated the Touch Experiences and Attitudes Questionnaire (TEAQ), which comprehensively assesses both past and current experiences of attitudes toward interpersonal touch and attitudes toward self-care^19^. Additionally, we focused on three subscales of TEAQ-G: retrospectively reported childhood caregiver touch, along with current attitudes toward and experiences of intimate touch. We explored how these subscales relate to attachment styles, social relationships, and mental health, as well as to individuals’ daily emotional states and hormonal levels (specifically cortisol and oxytocin, measured in daily life. While early caregiver tactile interactions are known to influence the development of brain regions involved in stress regulation^5^ less is known about how childhood touch experiences shape well-being and hormonal responses in adulthood and whether these effects persist over time. Similarly, little research has examined how attitudes toward touch moderate the relationship between affectionate touch, emotional well-being, and hormonal responses. Given the reported stress-reducing and beneficial effects of affectionate touch^20,9^, we expect these effects to be particularly pronounced in individuals with a more positive attitude toward intimate touch.

## Methods

### Participants and data collection

Data were obtained for this study from two different sources. To validate the German version of the TEAQ (TEAQ-G) and explore its associations with social relationships, mental health aspects, emotional and hormonal states, we initially obtained data from a larger longitudinal study focused on the psychobiological burden during the COVID-19 pandemic. Participants completed an online survey and were invited to participate in an additional two-day psychobiological Ecological Momentary Assessment^21,17,57^. Data collection took place between April and July 2021 (Dataset 1). Participants were recruited through various channels, including the university homepage, social media, and flyers. The data presented in this manuscript are part of a larger study that received approval from the ethics committee of Heidelberg University Medical Faculty (approval no. S-214/2020), in accordance with the Declaration of Helsinki. This study was registered online at the German Clinical Trials Register on 06/05/2020 with a clinical trial number DRKS00021671, which can be accessed at https://drks.de/search/de/trial/DRKS00021671 (accessed on 29.10.2024). The registration occurred immediately after the start of data collection due to the urgent implementation required by the onset of the pandemic, without preregistration of detailed hypotheses. All participants provided written informed consent.

To ensure a representative sample for TEAQ-G validation, we collected additional data through the online recruiting platform Clickworker (Dataset 2). To minimize participants’ time and effort, only a limited number of questionnaires were included in this online survey. We employed stratified sampling to target different age groups through the recruiting platform, aiming for a diverse distribution of participants. Data for this subsample was collected between August 2022 and October 2022. Eligibility criteria included a minimum age of 18 years, providing signed informed consent, and fluency in German.

**Fig. 1:**
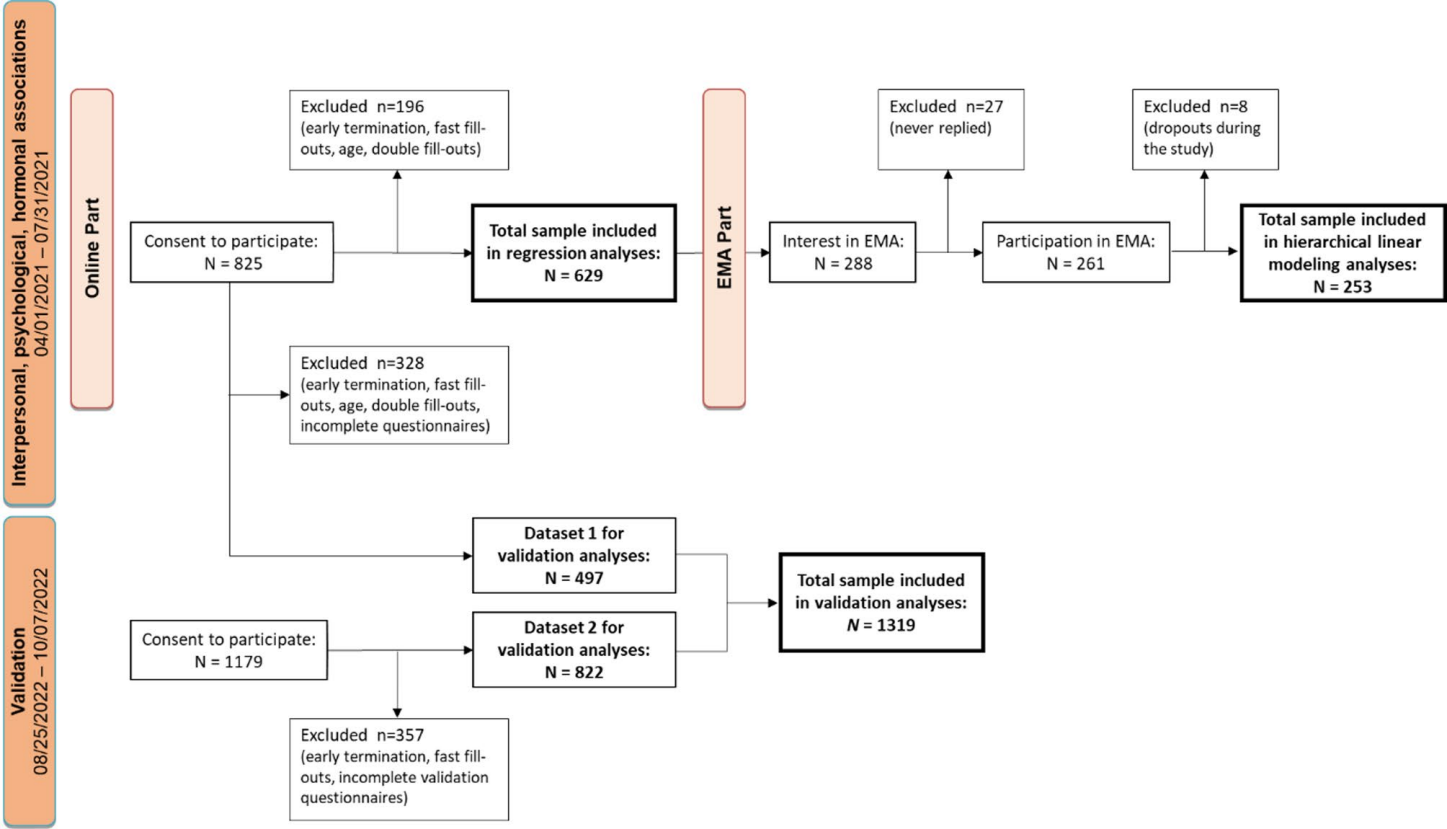
Flowchart of the recruitment process. Figure 1 illustrates the recruitment stages for both data sources. Participants were recruited for online participation and an additional 2-day Ecological Momentary Assessment (EMA) between April 1st and July 31st, 2021. Data from 629 participants were included in the regression analyses, while data from 253 individuals were analyzed from the EMA part. Additional data (Dataset 2) were collected through the Clickworker platform to validate the TEAQ-G, resulting in 1,319 individuals being included in validation analyses.

A total of 1,319 participants were included for TEAQ-G validation (for a detailed description, see Fig. 1). Among them, 644 individuals (48.8%) identified as female, 660 (50.0%) as male, 10 identified as diverse, and 5 chose not to disclose their gender. Of all participants, 838 (63.6%) were in a current relationship, 428 (32.5%) were single, 51 (3.9%) were divorced or widowed, and 2 participants did not report their relationship status. Participants’ age ranged from 18 to 81 years, with a mean age of 37.41(SD = 13.96).

The associations between TEAQ-G and social relationships and mental health aspects were analyzed in a subsample of 629 individuals. Among them, 494 (78.5%) identified as female, 129 (20.5%) as male, 3 identified as diverse, and 3 did not disclose their gender. The mean age of this sample was 34.8 (SD = 14.50). In our Ecological Momentary Assessment (EMA) study, which included hormonal measurements to evaluate hormonal states along with daily mental health aspects, 178 (70.4%) females, 74 (29.2%) males, and 1 individual without a specified gender participated, with a mean age of 34 (SD = 13.18) and ages ranging from 19 to 79 years (for detailed descriptive characteristics of the samples see Appendix 2).

### Psychological measures

In line with the original publication of Trotter et al.^19^, for the validation analyses of TEAQ-G, a Confirmatory Factor Analysis was conducted with a parceled model. Parcel-level modeling can improve indicator reliability, reduce sampling error on the item level, and achieve a more parsimonious model structure. This approach was implemented following the recommendations of^22,23^. Furthermore, we tested the association of its subscales with established questionnaires measuring touch attitude (the Social Touch Questionnaire (STQ))^24^, traumatic childhood experience (the Childhood Trauma Questionnaire (CTQ))^25^, and current level of social support (the Social Support Questionnaire (FsozU))^26^. To examine the relevance of touch experiences and attitudes in social relationships and mental health aspects, we used several measures, including the Experience in Close Relationships Scale (ECR) to measure attachment style^27^, Partnership Questionnaire (PFB) to measure relationship quality^28^, Systemic Clinical Outcome and Routine Evaluation 15 (SCORE-15), which assessed crucial and clinically significant aspects of family life^29^, General Trust Scale (GTS)^30^, Hospital Anxiety and Depression Scale (HADS)^31^, UCLA Loneliness Scale^32^, Perceived Stress Scale (PSS)^33^, and Brief Resilience Scale (BRS)^34^.

####  TEAQ

The Touch Experiences and Attitudes Questionnaire (TEAQ) consists of 57 items assessing six subscales. Three of these subscales focus on types of touch experiences: Childhood touch (ChT), current intimate touch (CIT), and family and friends touch (FFT). The other three subscales examine attitudes towards touch, including attitude to intimate touch (AIT), attitude to unfamiliar touch (AUT), and attitude to self-care (ASC). Examples are “There was a lot of physical affection during my childhood” (ChT), “I often share a romantic kiss” (CIT), “ I like it when my friends and family greet me by giving me a hug” (FFT), “Snuggling up on the sofa with someone is great” (AIT), “I am put off by physical familiarity” (AUT), “I like using body lotions” (ASC). Each item is rated on a 5-point Likert scale, where 1 indicates “Disagree strongly” and 5 indicates “Agree strongly”. Higher scores indicate more positive attitudes towards and experience with touch. The original version demonstrated good reliability and validity^19^, which we aimed to replicate in our German version. For example, the reliability of the subscales ranged from Cronbach’s alpha of α = 0.81 for the ASC to α = 0.93 for the CIT^19^. Two German speakers translated the items into German to create the German version of TEAQ (TEAQ-G). These translations were then back-translated into English. The resulting German version was reviewed by comparing it to the original TEAQ alongside the back-translated version. The team discussed and adjusted the items until they agreed on the precise wording. The final TEAQ-G version and the original version of TEAQ can be found in Appendix 1a and Appendix 1b respectively.

####  STQ

The validated German version of the Social Touch Questionnaire (STQ) consists of 20 items that measure the (dis)liking of different touch situations^24^. The STQ assesses various aspects of social touch, including touch with family and friends versus touch with strangers, touch occurring in different settings, and touch that has sexual connotations versus touch that does not. Exemplary items are “I hate being tickled.” (recoded), or “I feel comfortable touching people I don’t know well.” Participants respond to each item using a 5-point Likert scale, where 0 means “do not agree at all” and 4 means “agree completely”. Low scores on the STQ suggest a strong preference for social touch, while high scores indicate a high aversion to it. Cronbach’s α in our sample was α = 0.86.

####  FSozU

The Social Support Questionnaire is a short instrument originally developed in German. The questionnaire consists of 14 items that assess social support^26^. Statements of the items, e.g. “There are people who accept me for who I am without reservation.”, are rated on a 5-point Likert scale ranging from 1 “does not apply at all” to 5 “applies completely”. Higher scores indicate higher social support. Cronbach’s α in our sample was α = 0.94.

####  CTQ

The Childhood Trauma Questionnaire (CTQ) is an instrument to assess traumatic childhood experiences retrospectively. The validated German version of the CTQ with 25 items was used in this study^25^. The questionnaire comprises five subscales (emotional abuse, physical abuse, sexual abuse, emotional neglect and physical neglect), with five items per subscale. Examples are “When I was growing up, I was beaten so badly by someone in my family that I had to go to the doctor or hospital” (physical abuse), or “Growing up, I had to wear dirty clothes.” (physical neglect). Items are rated on a 5-point Likert scale ranging from 1 “never true” to 5 “very often true”. Higher scores suggest greater severity of traumatic experiences. Cronbach’s α was α = 0.88 in the given sample.

####  ECR

The Experience in Close Relationships Scale (ECR) is an instrument designed to assess adult attachment, originally consisting of 36 items^35^. Several brief versions of this scale have been developed and validated, including translations like the German short version of the Experiences in Close Relationships-Revised questionnaire (ECR-RD8)^27^. The questionnaire is divided into two subscales, each containing four items: attachment avoidance (e.g., “I find it easy to be affectionate towards my partner.”, recoded) and attachment anxiety (e.g., “I often worry that my partner doesn’t want to stay with me.”). Responses are provided on a 7-point Likert scale, ranging from 1 “do not agree at all” to 7 “agree completely”. Higher scores indicate greater discomfort and fear in close relationships, therefore stronger anxiety and avoidance. In the sample used for this study, the internal consistency was measured with a Cronbach’s α of 0.79.

####  PFB

The short version of the Partnership Questionnaire (Partnerschaftsfragebogen, PFB) is a German questionnaire assessing relationship quality^28^. The questionnaire comprises 10 items examining three subscales: quarreling (e.g., “He/she makes derogatory remarks about an opinion I have expressed.”, tenderness (e.g., “He/she caresses me tenderly.”, and communication (e.g., “We talk to each other for at least half an hour in the evening.”). Answers are given on a 4-point Likert scale ranging from 0 “never” to 3 “very often”, for the last item on a 6-point Likert scale ranging from 0 “very unhappy” to 3 “very happy”. After recoding the quarreling subscale, higher values of the PFB represent higher self-reported relationship quality. Cronbach’s α was α = 0.88 in our sample.

####  SCORE-15

The short version of Systemic Clinical Outcome and Routine Evaluation 15 (SCORE-15) is a measure used to assess family functioning^29^. It consists of 15 items divided into three subscales: (1) Strengths and Adaptability, (2) Overwhelmed by Difficulties, and (3) Disrupted Communication. Each subscale contains five items. Responses are rated on a 5-point Likert scale, where 1 indicates “describes us very well” and 5 indicates “describes us not at all.” Lower total scores indicate high functioning. Exemplary items are “In my family, we discuss things that are important to us.”, or “Everyone in our family is listened to.”. Cronbach’s α was α = 0.92 in our sample.

####  GTS

The General Trust Scale assesses the general trust level in other people when there is insufficient information about their trustworthiness^30^. This scale comprises six items, with responses ranging from 1 “strong disapproval” to 5 “strong approval” on a 5-point Likert scale. Exemplary items are “Most people are basically honest.”, or “I am trustful.”. Higher scores indicate higher levels of generalized trust. In our sample internal consistency of Cronbach’s α = 0.84 could be reported.

####  HADS

In our study, we used the German version of the Hospital Anxiety and Depression Scale (HADS) to assess anxiety and depression^31^. The scale consists of two subscales, each containing seven items, which participants answer using a 4-point Likert scale ranging from 0 to 3. Exemplary items are “I feel held back in my activities.” (depression subscale), or “I suddenly feel panic coming over me.” (anxiety subscale). Higher sum scores represent higher levels of depression and anxiety. Internal Consistency in our sample was Cronbach’s α = 0.89.

####  UCLA Loneliness scale

The German version of the UCLA Loneliness Scale is a general measure of loneliness (e.g., “How often do you feel that you are no longer close to anyone?”), which respondents answer using a 4-point Likert scale, ranging from 1 “never” to 4 “often”^32^. Higher values on the scale indicate higher levels of loneliness. In our sample, we found an internal consistency of α = 0.92.

####  PSS

The Perceived Stress Scale (PSS) is a tool used to assess how individuals perceive stressful situations. While the original version contains 14 items, the German 10-item version is more commonly used^33^. Respondents answer each item using a 5-point Likert scale, ranging from 1 “never” to 5 “very often”. Exemplary items are “How often did you feel in control of everything in the last month?” (recoded), or “How often did you feel nervous and ‘stressed’ in the last month?”. Higher values suggest a higher level of perceived stress. In this sample, the internal consistency was α = 0.90.

####  BRS

The Brief Resilience Scale (BRS) is a short self-report measure to assess resilience, defined as one’s ability to recover from stress^34^. The questionnaire consists of six items, and responses are provided on a 5-point Likert scale, ranging from 1 “do not agree at all” to 5 “agree completely”. Items are, e.g., “I tend to bounce back quickly after hard times” or “It is hard for me to snap back when something bad happens.” (recoded). Higher scores indicate greater resilience. In our sample, the internal consistency was high, with a Cronbach’s alpha of α = 0.87.

### Measures of psychobiological ecological momentary assessment (EMA)

Participants interested in EMA participation received phone instructions on how to use their smartphones to collect momentary subjective data and saliva samples through passive drool. Over two consecutive days, participants provided a total of 12 saliva samples at six time points each day, scheduled according to their wake-up times: immediately after waking, 30 min later, 45 min later, 2.5 h later, 8 h later, and just before going to sleep.

Simultaneously, participants completed subjective ratings, answering questions about their current emotional state, including happiness, stress, anxiety, loneliness, and the burden related to the current COVID-19 pandemic. These items were assessed using visual analog scales ranging from 0 (not at all) to 100 (very much). At each time point, participants indicated whether they had experienced affectionate touch since the last time point and specified the type of touch they experienced, such as hugs, cuddles, caresses, kisses, or sexual activity. The items were presented in the same order. Compliance with data collection was monitored online, and phone reminders were sent if participants did not access the link within five minutes. Response rates were high, with percentages ranging from 98.75% for momentary loneliness data to 99.41% for both stress and happiness. After the two days of sampling, the data were stored on a university internal server, while the saliva samples remained in participants’ freezers until collection.

The samples were collected from the participants’ homes and were stored at -80 °C and analyzed at the biochemical lab of the Institute of Medical Psychology at Heidelberg University Hospital. The analysis of oxytocin concentrations was completed without extraction, with 50% of the samples analyzed in duplicates. We followed the protocol for the oxytocin enzyme-linked immunosorbent assay (ELISA) from Enzo Life Sciences (Switzerland), which has a detection limit of 15 pg/ml. In our sample, the variation coefficient for intra-assay precision was 5.9%, while the inter-assay precision was 13.63%. Cortisol levels were analyzed, with 20% of the samples in duplicates using an ELISA from Demeditec Diagnostics (Germany), with a detection limit of 0.019 ng/ml. Intra-assay and inter-assay variations in our sample were 2.8% and 5.9%, respectively. These variation coefficients represent good intra- and inter-assay precision, with values lower than 10% and 15%, respectively^36^.

### Statistical analyses

#### Validation of the TEAQ-G

Data preprocessing was carried out using IBM SPSS version 27 and R studio (version 2024.12.0.467). Following the procedure of the original validation study^19^, we performed confirmatory factor analysis (CFA) using AMOS statistical software (Amos™ 7; SPSS Inc.) to examine the structure of the TEAQ-G in our sample. As shown by the original TEAQ version, a factor structure with six components was expected. The goodness of the model fit was evaluated by the Root Mean Square Error of Approximation (RMSEA) < 0.06 with a 90% confidence interval, Standardized Root Mean Square Residual (SRMR) < 0.05, the Comparative Fit Index (CFI) > 0.95, and the Tucker-Lewis Index (TLI) > 0.95^37^. As suggested by the original validation paper, a parceled model was applied to improve the model fit^19^. With parceling, multiple variables or items are combined into a single aggregated parcel to reduce the number and complexity of indicators. In the case of the TEAQ-G, 3 parcels were calculated for each component, each with an approximately equal number of items. Since the components consisted of unequal numbers of items, not all parcels contained the same number of variables. The parceling procedure was as balanced as possible, as the lowest and highest loading items were parceled together to ensure similar loading of each parcel onto its latent variable. To compare the original and parceled model, Akaike’s Information Criterion (AIC) was assessed, in addition to the other fit indices.

The reliability of the TEAQ-G was determined by calculating Cronbach’s α as well as the discriminatory power as item-total correlation. The criterion-related validity was assessed by correlating the TEAQ-G with the following three questionnaires: For concurrent validity, all subscales of TEAQ-G were associated with another, previously published Social Touch Questionnaire (STQ)^24^. In line with the original TEAQ validation paper^19^, we correlated the TEAQ-G subscales with the Childhood Trauma Questionnaire (CTQ)^25^ and the Social Support Questionnaire (FSozU)^26^ to assess predictive validity.

#### The role of TEAQ-G in social relationships and mental health aspects

To further investigate the the link between TEAQ-G and social relationships and mental health, we conducted multiple regression analyses using SPSS. We assessed the general assumptions for regression models, including linearity, homoscedasticity, independence of error terms, and multicollinearity, and found no violations of these assumptions.

In separate models, we included scores of social relationships (ECR anxiety, ECR avoidance, PFB, GTS, Score-15) and mental health aspects (HADS, UCLA Loneliness, PSS, BRS) as dependent variables. The independent variables in each model consisted of the subscales of TEAQ-G (FFT, CIT, AUT, ChT, AIT, ASC) while controlling for age, gender, and relationship status. For simplicity and due to the small number of cases, gender (male vs. female) and relationship status (single vs. in a relationship) were dummy-coded. Although our main interest was in three specific subscales of TEAQ-G (ChT, CIT, AIT), we included all six to account for shared variance among them. To control for multiple comparisons in our regression models, we applied a Bonferroni correction, resulting in a significance threshold of α = 0.01 for social relationship outcomes (5 models) and α = 0.013 for mental health outcomes (4 models).

To analyze whether our models were adequately powered, we conducted a post-hoc power analysis using G*Power^38^.

#### Affectionate touch, TEAQ-G, and individuals’ emotional and hormonal state in everyday life

We conducted hierarchical linear models to examine the relationship between daily affectionate touch, touch attitudes and experiences measured by the TEAQ-G, and individuals’ emotional and hormonal states as indicators of psychobiological well-being. In the first set of analyses, we included daily mean values of hormonal levels (cortisol, oxytocin) and individuals’ emotional states - specifically stress, anxiety, loneliness, happiness, and COVID-19-related burden as outcome variables, using the subscales of the TEAQ-G as predictor variables. In subsequent analyses, we assessed the individuals’ momentary emotionall states alongside hormonal levels. Here, we used momentary affectionate touch levels (summed from self-reported types of touch at each time point), the TEAQ-G AIT, and its interaction variable as predictors. To separate within-person and between-person effects, we centered the self-reported momentary affectionate touch variable around each individual’s mean, while also centering each person’s mean around the grand mean. All models controlled for age, gender, relationship status, and measuring day. For models with cortisol and oxytocin levels as the outcome variables, we additionally controlled for body mass index (BMI) and a range of potential confounding factors, such as momentary food and drink intake, caffeine and cigarette consumption, and physical activity. Furthermore, we accounted for assessment time points by including time (coded from 0 to 3 for the assessment time points 3 to 6) to control for linear diurnal changes after waking^39^. Before analyses, cortisol and oxytocin levels were log-transformed (natural logarithm) to normalize the distribution. In line with recent recommendations^40^, we did not apply alpha corrections to our multilevel analyses. Each model tested predictors on distinct, single-item outcomes, which do not form a shared construct or omnibus hypothesis. Each test addresses a separate aspect of momentary emotional and hormonal state.”

## Results

### Validation of TEAQ-G questionnaire

The results of the confirmatory factor analysis for the original model, with all items included separately, showed a rather moderate fit. However, the model fit improved significantly through parceling, which was consistent with the original validation^19^. All fit indices for the parceled model met the required criteria (CFI = 0.969; RMSEA = 0.058, 90%CI = 0.054-0.063; SRMR = 0.0387) (see Table 1a), suggesting that the 6-factor structure can be considered confirmed. Since data included two different sample collection sources, both subsamples were also analyzed separately to check for systematic differences due to the collection source. The confirmatory factor analysis was conducted in both subsamples as well using the parceled model, indicating comparable and satisfactory model fit in both datasets (Dataset 1: CFI = 0.957; RMSEA = 0.068; SRMR = 0.049; versus Dataset 2: CFI = 0.975; RMSEA = 0.053; SRMR = 0.037). Consequently, we used the whole sample for further validation analyses. The reliability analysis showed that internal consistency was good in our sample ranging from Cronbach’s α = 0.80 for the attitude to self-care factor (ASC) to Cronbach’s α = 0.93 for the “current intimate touch” factor (CIT) (see Table 1b). The discriminatory power of all items was within the recommended range of 0.4 < *r*_*itc*_ < 0.7^41^ or slightly above, varying from *r*_*itc*_=0.471 (item 42) to *r*_*itc*_= 0.788 (item 41). For criterion-related validity, Spearman’s rho correlations showed that another validated social touch questionnaire (STQ) (low scores indicate a positive attitude towards touch) significantly moderately to strongly correlated with all TEAQ-G subscales (ranging from ρ= − 0.44 to ρ= − 0.75, *p < .*01), except for ASC (ρ=-0.22, *p* < .01). Similarly, all TEAQ-G scales significantly positively correlated with perceived social support as measured with FSozU (ρ= − 0.198 to ρ= − 0.523, *p < .*001). Furthermore, childhood trauma (CTQ) showed significantly negative correlations with all TEAQ-G scales (ranging from ρ=-0.130 to ρ= − 0.644, *p < .*001), except for ASC (ρ = − 0.011, *p = .*716) (see Table 1c).

**Table 1:** Validation of TEAQ-G through confirmatory factor analysis, internal consistency, and criterion-related validity.

a) Model fit indices for the TEAQ-G models tested using confirmatory factor analysis						
	Goodness of fit tests (criterion value)					
	CFI (> 0.95)	TLI (> 0.95)	RMSEA (< 0.06) 90% CI	SRMR (< 0.05)	AIC	
Original model	0.754	0.742	0.075 0.074-0.076	0.07	13166.892	
Parceled model	0.969	0.961	0.058 0.054-0.063	0.039	762.87	

b) Internal consistency of TEAQ-G components						
	FFT	CIT	ChT	ASC	AIT	AUT
Cronbach’s α	0.90	0.93	0.91	0.80	0.91	0.81

c) Spearman’s Rho correlations of TEAQ and other touch-related instruments						
Spearman’s ρ	STQ		FsozU		CTQ	
TEAQ-G ChT	− 0.45**		0.44**		− 0.64**	
TEAQ-G CIT	− 0.44**		0.52**		− 0.25**	
TEAQ-G FFT	− 0.58**		0.43**		− 0.13**	
TEAQ-G AIT	− 0.57**		0.42**		− 0.20**	
TEAQ-G AUT	− 0.75**		0.27**		− 0.18**	
TEAQ-G ASC	− 0.22**		0.20**		− 0.01	

Table 1 summarizes the results from the validation analyses of TEAQ-G. RMSEA = Root Mean Square Error of Approximation; SRMR = Standardized Root Mean Square Residual; CFI = Comparative Fit Index; TLI = Tucker-Lewis Index; AIC = Akaike’s Information Criterion; TEAQ-G = Touch Experiences and Attitudes Questionnaire German; ChT = TEAQ Childhood Touch; CIT = TEAQ Current Intimate Touch; FFT = TEAQ Friends and Family Touch; AIT = TEAQ Attitude to Intimate Touch; AUT = TEAQ Attitude to Unfamiliar Touch; ASC = TEAQ Attitude to Self-Care; STQ = Social Touch Questionnaire; FsozU = Social Support Questionnaire; CTQ = Childhood Trauma Questionnaire.

Abbr. outcomes. ^*^p < .05. ^**^p < .01. (2-tailed).

Correlation coefficients > 0.3 are indicated in bold.

Since our sample covered a broad age range, we took the opportunity to explore the relationship between TEAQ-G subscales and age. A MANOVA revealed that age had a significant moderate overall effect on the combined TEAQ-G subscales, Pillai’s Trace = 0.10, F(6,1311) = 24.37, *p* < .001, partial η² = 0.10. Follow up univariate tests showed that age was significantly and positively associated with friends and family touch (TEAQ-G FFT; *ß* = 0.06,t = 2.16, *p* = .031, partial η² = 0.004) but negatively with current intimate touch (TEAQ-G CIT; *ß* = −0.15, t = .-4.53, *p* < .001, partial η² = 0.015). Additionally, older participants reported significantly fewer childhood touch experiences (TEAQ-G ChT; *ß* = −0.15, t= -5.61, *p* < .001, partial η² = 0.023). Regarding the relationship between age and attitudes toward touch, we found that higher age was associated with less positive attitudes toward unfamiliar touch (TEAQ-G AUT; *ß* = −0.14, t= -5.18, *p* < .001, partial η² = 0.020). Interestingly, attitudes toward intimate touch (TEAQ-G AIT; *ß* = −0.01, t = − 0.39, *p* = .700) and self-care touch (TEAQ-G ASC; *ß* = −0.05, t = -1.86, *p* = .064) did not show significant associations with age. Overall effect sizes were small throughout the analyses.

### The role of TEAQ-G in social relationships and mental health aspects

All results of the regression analyses are summarized in supplementary Table (Appendix 3). Our primary focus is on the impact of touch experiences during development (childhood and adulthood) and attitudes toward intimate touch. For this reason, we selectively present the findings related to the TEAQ-G ChT, TEAQ-G CIT, and TEAQ-G AIT measures in the following text section and Table 2. The reported p-values are unadjusted; however, statistical significance was evaluated using a Bonferroni-corrected threshold (*p* < .01 for social relationship outcomes; *p* < .013 for mental health outcomes).

First, we analyzed the association of TEAQ-G subscales with the questionnaires designed to evaluate social relationships. Our analyses revealed that retrospectively reported touch experiences during childhood (TEAQ-G ChT) significantly and negatively predicted current attachment avoidance (ECR avoidance) (β=-0.13, *T*=-3.016, *p* = .003) but positively predicted general trust (GTS) (β = 0.13, *T* = 2.664, *p* = .008). Furthermore, childhood touch experiences were negatively correlated with family functioning (Score-15), (β=-0.37, *T*=-8.162, *p* < .001), indicating that higher childhood touch experiences were related to higher levels of family functioning. Current intimate touch (TEAQ-G CIT) was significantly and negatively associated with attachment anxiety (ECR anxiety) (β=-0.523, T=-7.370, *p* < .001), attachment avoidance (ECR avoidance) (β=-0.339, *T*=-5.165, *p* < .001), and family functioning (Score-15) (β=-0.344, *T*=-5.089, *p* < .001). In the subsample of participants who were in a romantic relationship, TEAQ-G CIT was significantly and positively associated with relationship quality (PFB) (β = 0.68, *T* = 10.690, *p* < .001). Attitude toward intimate touch (TEAQ-G AIT) positively predicted attachment anxiety (ECR anxiety) (β = 0.22; *T* = 3.806, *p* < .001), and family functioning (Score-15) (β = 0.18, *T* = 3.163, *p* = .002), but was negatively associated with attachment avoidance (ECR avoidance) (β=-0.24, *T*=-4.372, *p* < .001) and relationship quality (PFB) (β=-0.21, *T*=-3.438, *p* = .001).

Next, we analyzed how TEAQ-G predicted individuals’ mental health aspects and found that touch experience during childhood (TEAQ-G ChT) negatively predicted anxiety and depression (HADS) (β=-0.21, *T*=-4.47, *p* < .001), loneliness (UCLA Loneliness Scale) (β=-0.19, *T*=-4.40, *p* < .001), and stress (PSS) (β=-0.42, *T*=-5.92, *p* < .001), but positively predicted resilience (BRS) (β = 0.16, *T* = 3.34, *p* < .001) (see Table 2). Similarly, current intimate touch (TEAQ-G CIT) was negatively associated with anxiety and depression (HADS) (β=-0.47, *T*=-6.59, *p* < .001), loneliness (UCLA Loneliness Scale) (β=-0.60, *T*=-9.29, *p* < .001), and stress (PSS) (β=-0.42, *T*=-5.92, *p* < .001), but positively associated with resilience (BRS) (β = 0.34, *T* = 4.54, *p* < .001). Attitude toward intimate touch (TEAQ-G AIT) was positively associated with loneliness (UCLA Loneliness Scale) (β = 0.22, *T* = 4.20, *p* < .001).

Additionally, we conducted a post-hoc power analysis for regression analyses to ensure that the available sample size (*N* = 629), the alpha level of 5%, and the nine predictors (six TEAQ-G subscales and three demographic controls) provided sufficient statistical power. The analysis indicated that the achieved power was 99% for detecting even the smallest effect (GTS scale) with a critical F-value of 1.89, confirming that the models were adequately powered.

**Table 2:** Results of regression analyses with TEAQ-G (CIT, ChT, AIT) predicting outcomes of social relationships and mental health aspects.

Outcome		Social relationships					Mental health aspects			
		ECR anxiety	ECR avoidance	PFB	Score-15	GTS	HADS	UCLA Loneliness	PSS	BRS
Constant	b (SE; *p*)	16.25 (1.82; <0.001)	22.32 (1.45; <0.001)	16.61 (1.86; <0.001)	3.51 (0.21; <0.001)	2.59 (0.20; <0.001)	27.67 (2.34; <0.001)	63.42 (3.19; <0.001)	41.61 (2.50; <0.001)	2.29 (0.26; <0.001)
ChT	β (SE; *p*)	− 0.05 (0.28; 0.256)	**− 0.13** **(0.22; 0.003)**	− 0.01 (0.28; 0.847)	**− 0.37** **(0.03; <0.001)**	**0.13** **(0.03; 0.008)**	**− 0.21** **(0.36; <0.001)**	**− 0.19** **(0.49; <0.001)**	**− 0.18** **(0.39; 0.001)**	**0.16** **(0.04; 0.001)**
CIT	β (SE; p)	**− 0.52** **(0.44; <0.001)**	**− 0.34** **(0.35; <0.001)**	**0.68** **(0.43; <0.001)**	**− 0.34** **(0.05; <0.001)**	0.08 (0.05; .25*1*)	**− 0.47** **(0.58; <0.001)**	**− 0.60** **(0.79; <0.001)**	**− 0.42** **(0.62; <0.001)**	**0.34** **(0.06; <0.001)**
AIT	β (SE; *p*)	**0.22** **(0.49; 0.002)**	**− 0.24** **(0.39; <0.001)**	**− 0.21** **(0.49; 0.001)**	**0.18** **(0.06; 0.002)**	− 0.04 (0.05; 0.499)	0.10 (0.64; 0.084)	**0.22** **(0.88; <0.001)**	0.14 (0.69; 0.015)	− 0.09 (0.07; 0.158)
**Model**	**R** ^**2**^ **(F;** ***p*)**	**0.19** **(12.203; <0.001)**	**0.31** **(23.288; <0.001)**	**0.38** **(25.853; <0.001)**	**0.26** **(19.044; <0.001)**	**0.10** **(6.189; <0.001)**	**0.20** **(13.202; <0.001)**	**0.33** **(26.780; <0.001)**	**0.17** **(11.212; <0.001)**	**0.13** **(7.715; <0.001)**

*N* = 344–527. Standardized coefficients (β), standard errors (SE), and p-values are displayed. Bold values indicate p-values significant at Bonferroni-adjusted α. Abbr. outcomes. FFT = Touch Experiences and Attitudes Questionnaire (TEAQ) Friends and Family Touch; CIT = TEAQ Current Intimate Touch; ChT = TEAQ Childhood Touch; ASC = TEAQ Attitude to self-care; AIT = TEAQ Attitude to Intimate Touch; AUT = TEAQ Attitude to Unfamiliar Touch; ECR = Experience in Close Relationships Scale; PFB = Partnership Questionnaire; Score-15 = Systemic Clinical Outcome and Routine Evaluation 15 (lower values indicate higher family functioning); GTS = General Trust Scale; HADS = Hospital Anxiety and Depression Scale; UCLA Loneliness = Loneliness Scale; PSS = Perceived Stress Scale; BRS = Brief Resilience Scale. ^a^ 0 = male, 1 = female. ^b^ 0 = no, 1 = yes.

### Affectionate touch, TEAQ-G, and individuals’ emotional and hormonal state in everyday life

The analyses of the relationship between the TEAQ-G subscales and emotional and hormonal states, based on Ecological Momentary Assessment data, revealed several noteworthy associations. Current Intimate Touch (TEAQ-G CIT) was found to be marginally negatively associated with aggregated levels of stress (*b*=-4.239; *t*(233)=-1.910; *p* = .057), pandemic-related burden (*b*=-7.497; *t*(233)=-2.531; *p* = .012), and loneliness (*b*=-9.672; *t*(233)=-4.014; *p* < .001). Conversely, it was positively associated with aggregated happiness (*b* = 8.769; *t*(233) = 4.089; *p* < .001) and salivary oxytocin levels (*b* = 0.142; *t*(222) = 1.924; *p* = .056). Additionally, the attitude towards intimate touch (TEAQ-G AIT) was significantly positively associated with loneliness (*b* = 9.195; *t*(233) = 3.139; *p* = .002) and showed a marginal association with pandemic-related burden (*b* = 6.548; *t*(233) = 1.776; *p* = .077). Notably, touch experiences during childhood (TEAQ-G ChT) significantly predicted various self-reported psychological states: stress (*b*=-4.945; *t*(233)=-3.701; *p* < .001), pandemic-related burden (*b*=-5.777; *t*(233)=-3.114; *p* = .002), anxiety (*b*=-5.245; *t*(233)=-3.526; *p* < .001), loneliness (*b*=-4.345; *t*(233)=-2.954; *p* = .004) and happiness (*b* = 4.042; *t*(233) = 3.104; *p* = .002) (see Table 3 (A) and Table 4 (A)).

**Fig. 2:**
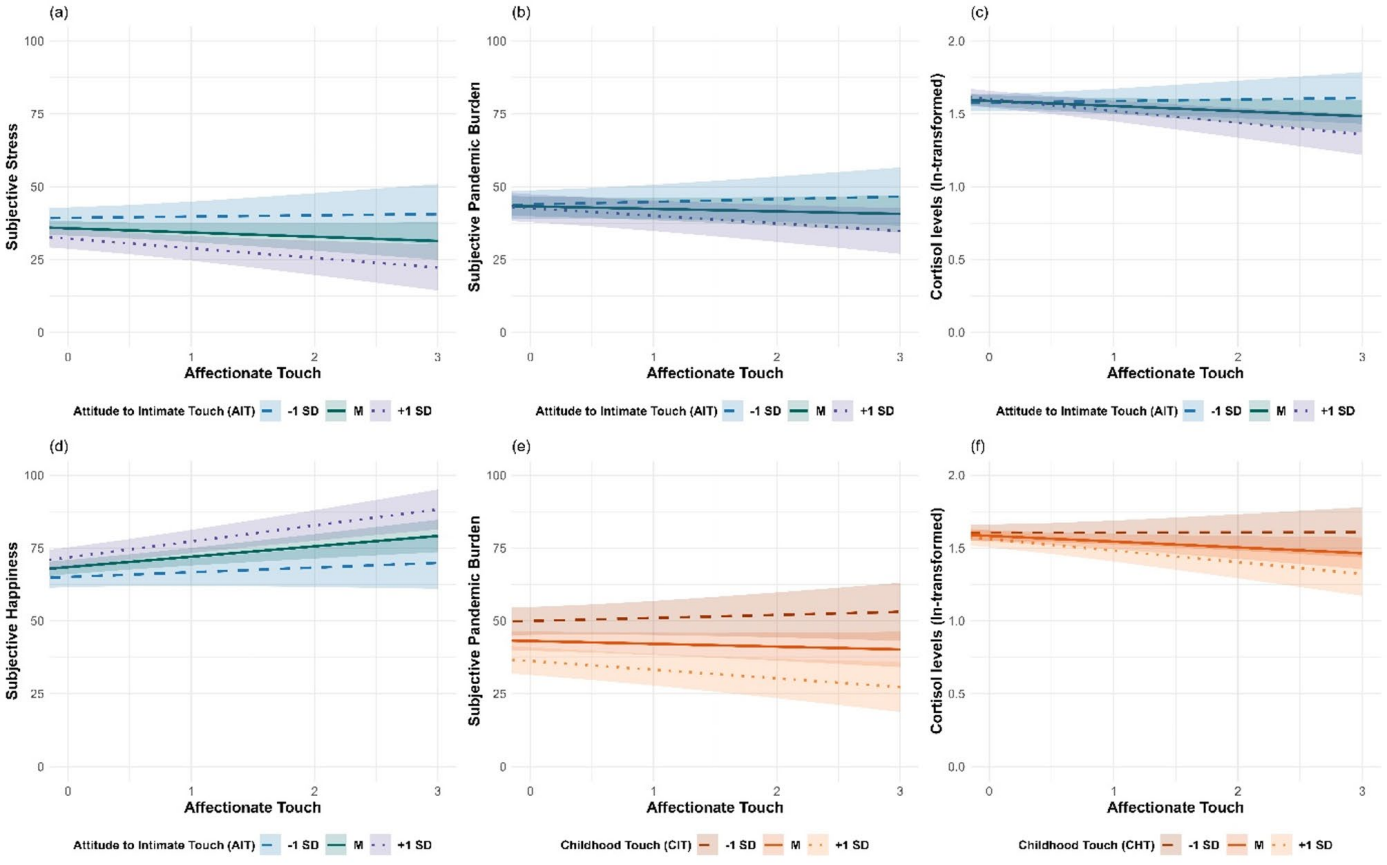
Attitude toward intimate touch and childhood touch experience moderate the associations between daily affectionate touch and emotional and hormonal states. Panels (**a**) to (**f**) illustrate significant moderations of attitude toward intimate touch (a-d) and childhood touch experiences (**e**–**f**) with daily reported affectionate touch predicting subjective ratings of stress, pandemic-related burden, happiness, and cortisol levels. Solid lines represent the mean, dashed lines represent one standard deviation below the mean (−1 SD), and dotted lines represent one standard deviation above the mean (+ 1 SD) of attitude toward intimate touch or childhood touch experience.

Next, we examined how momentary touch, TEAQ-G AIT, and their interaction predicted the individuals’ momentary emotional and hormonal states. Results from separate random intercept and slopes multilevel analyses showed that on a within-person level, momentary affectionate touch was significantly negatively associated with stress (*b*=-1.955; *t*(474)=-3.488; *p* < .001) and loneliness (*b*=-1.726; *t*(611)=-4.465; *p* < .001) while being positively associated with happiness (*b* = 1.955; *t*(474) = 4.456; *p* < .001). On a between-person level, we found a significant interaction between affectionate touch and attitude toward intimate touch (TEAQ-G AIT) predicting pandemic-related burden (*b*=-2.908; *t*(472)=-2.068; *p* = .039), happiness (*b* = 3.209; *t*(474) = 2.445; *p* = .015), cortisol levels (*b*=-0.078; *t*(568)=-2.714; *p* = .007), and stress (*b*=-3.096; *t*(474)=-1.998; *p* = .046). This interaction indicates that especially individuals with a very positive attitude towards touch show the expected association: more momentary touch is linked to lower burden, stress, and cortisol, and higher happiness. However, no significant interactions were observed in models predicting anxiety, loneliness, or oxytocin levels (see Table 3 (B), Table 4 (B), and Fig. 2 for illustration).

After we found that childhood touch experiences (TEAQ-G ChT) significantly predicted psychological well-being in daily life, we performed exploratory analyses to determine whether TEAQ-G ChT moderated the relationship between affectionate touch and individuals’ psychological and hormonal states. A significant interaction was found between affectionate touch and TEAQ-G ChT in predicting pandemic-related burden (*b*=-1.970; *t*(472)=-2.116; *p* = .035), and cortisol levels (*b*=-0.041; *t*(568)=-2.249; *p* = .025) and on a trend-level prediction for happiness (*b* = 1.463; *t*(474) = 1.711; *p* = .088) (see Table 3 (C), Table 4 (C), and Fig. 2). This interaction indicates that individuals, who retrospectively reported more positive childhood touch experiences exhibit the expected pattern: more momentary touch is linked to lower burden and cortisol levels, and shows a trend towards a positive association with happiness.

**Table 3:** Associations between experiences and attitudes towards touch (TEAQ-G scales), momentary touch and emotional states.

Effects	Stress	Covid-19 burden	Anxiety	Loneliness	Happiness
(A) Associations between experiences and attitudes towards touch (TEAQ-G) and emotionalstates					
Fixed effects					
Intercept	69.134 (1.074); p<.001	54.046 (13.972); p<.001	57.045 (11.207); p<.001	45.541 (11.084); p<.001	42.679 (9.812); p<.001
TEAQ AIT	.715 (2.659); p=.788	6.548 (3.686); p=.077	-2.513 (2.958); p=.397	**9.195 (2.926); p=.002**	-4.098 (2.590); p=.115
TEAQ ASC	.031 (1.287); p=.981	1.414 (1.786); p=.430	1.329 (1.433); p=.356	.121 (1.417); p=.932	.074 (1.254); p=.953
TEAQ AUT	-1.537 (1.445); p=.289	2.255 (2.006); p=.262	-.130 (1.609); p=.936	-.467 (1.591); p=.769	-.798 (1.408); p=.571
TEAQ ChT	**-4.945 (1.336); p<.001**	**-5.777 (1.855); p=.002**	**-5.245 (1.488); p<.001**	**-4.345 (1.471); p=.004**	**4.042 (1.302); p=.002**
TEAQ CIT	-4.239 (2.220); p=.057	**-7.497 (2.962); p=.012**	-.827 (2.427); p=.734	**-9.672 (2.409); p<.001**	**8.769 (2.144); p<.001**
TEAQ FFT	2.121 (1.933); p=.274	.409 (2.671); p=.879	1.323 (2.147); p=.538	.656 (2.124); p=.758	1.088 (1.882); p=.564
Covariates					
Age	**-.302 (.103); p=.004**	-.272 (.142); p=.057	** -.273 (.114); p=.018**	**-.350 (.113); p=.002**	.186 (.100); p=.064
Sex^b^	**7.593 (2.893); p=.009**	5.712 (4.015); p=.156	3.327 (3.220); p=.303	2.434 (3.184); p=.445	**-1.067 (2.819); p<.001**
Day^c^	**-2.248 (.985); p=.023**	**-3.144 (.719); p<.001**	**-2.222 (.762); p=.004**	**-1.862 (.804); p=.021**	**1.974 (.788); p=.013**
Partner^d^	.970 (3.451); p=.779	1.298 (4.258); p=.761	-1.935 (3.649); p=.596	-.338 (3.651); p=.926	-2.924 (3.284); p=.374
Random effects (SD)					
Intercept	9.687	6.582	7.223	7.672	7.602
Residual	4.819	4.354	4.228	4.366	4.134

(B) Associations between attitude to intimate touch (TEAQ-G AIT), momentary touch and emotional states					
Fixed effects					
Within-person					
Intercept	48.803 (5.512); p<.001	57.508 (6.765); p<.001	26.641 (5.671); p<.001	36.051 (5.273); p<.001	68.418 (5.118); p<.001
Touch^a^	**-1.955 (.561); p<.001**	-.034 (.440); p=.938	-.455 (.459); p=.322	**-1.726 (.387); p<.001**	**1.955 (.439); p<.001**
Between person					
Touch^a^	-1.299 (1.001); p=.195	-.722 (.879); p=.412	-1.118 (.890); p=.209	**-3.774 (.806); p<.001**	**3.406 (.838); p<.001**
TEAQ AIT	**-5.891 (2.135); p=.006**	-1.044 (2.882); p=.718	**-5.103 (2.314); p=.028**	-.634 (2.310); p=.784	**5.538 (2.079); p=.008**
Touch^a^*TEAQ AIT	**-3.096 (1.549); p=.046**	**-2.908 (1.406); p=.039**	-.731 (1.396); p=.601	-.644 (1.306); p=.622	**3.209 (1.312); p=.015**
Covariates					
Age	-.121 (.101); p=.232	-.048 (.138); p=.727	-.137 (.110); p=.213	-.198 (.108); p=.069	.080 (.099); p=.418
Sex^b^	**6.267 (2.737); p=.023**	3.885 (3.736); p=.299	4.004 (2.987); p=.181	-1.367 (2.977); p=.647	**-6.604 (2.679); p=.014**
Day^c^	-1.349 (1.026); p=.189	**-2.432 (.138); p=.002**	-1.200 (.842); p=.155	**-1.653 (.746); p=.027**	1.541 (.804); p=.056
Partner^d^	-2.131 (3.239); p=.511	-4.716 (4.198); p=.262	.364 (3.423); p=.915	-6.280 (3.395); p=.065	1.884 (3.094); p=.543
Time^e^	**-2.235 (.538); p<.001**	**-2.148 (.456); p<.001**	-.394 (.446); p=.378	**1.180 (.233); p<.001**	-.134 (.421); p=.751
Random effects (SD)					
Intercept	0.003	0.006	0	0	0.001
Time^e^	<.001	<.001	<.001	<.001	<.001
Residual	17.509	13.304	14.294	14.736	13.68

(C) Associations between retrospectively reported childhood touch (TEAQ ChT), momentary touch and emotional states					
Fixed effects					
Within person					
Intercept	49.985 (5.392); p<.001	59.674 (6.662); p<.001	28.887 (5.556); p<.001	38.456 (5.140); p<.001	67.242 (4.986); p<.001
Touch^a^	**-1.948 (.561); p<.001**	-.037 (.439); p=.933	-.456 (.460); p=.321	**-1.724 (.386); p<.001**	**1.949 (.439); p<.001**
Between person					
Touch^a^	-1.657 (.961); p=.086	-.887 (.845); p=.295	-1.209 (.856); p=.159	**-4.041 (.776); p<.001**	**3.777 (.805); p<.001**
TEAQ ChT	**-6.020 (1.265); p<.001**	**-6.743 (1.719); p<.001**	**-5.545 (1.377); p<.001**	**-4.908 (1.368); p<.001**	**6.107 (1.227); p<.001**
Touch^a^*TEAQ ChT	-1.312 (.997); p=.189	**-1.970 (.931); p=.035**	-.175 (.916); p=.849	.828 (.845); p=.328	1.463 (.855); p=.088
Covariates					
Age	-.197 (.100); p=.052	-.181 (.138); p=.191	**-.223 (.110); p=.043**	**-.325 (.108); p=.003**	.160 (.098); p=.104
Sex^b^	**7.422 (2.661); p=.006**	5.189 (3.664); p=.158	**4.901 (2.916); p=.094**	-.462 (2.894); p=.873	**-7.761 (2.595); p=.003**
Day^c^	-1.353 (1.028); p=.189	**-2.476 (.789); p=.002**	-1.198 (.842); p=.156	**-1.613 (.747); p=.031**	1.545 (.806); p=.056
Partner^d^	-1.761 (3.097); p=.570	-2.972 (4.060); p=.463	.226 (3.284); p=.945	-4.890 (3.245); p=.132	1.340 (2.948); p=.650
Time^e^	**-2.241 (.536); p<.001**	**-2.158 (.455); p<.001**	-.392 (.446); p=.380	**1.176 (.233); p<.001**	-.123 (.420); p=.770
Random effects (SD)					
Intercept	0.003	0.007	0.001	0	0.001
Time^e^	<.001	<.001	<.001	<.001	<.001
Residual	17.537	13.287	14.3	14.734	13.705

Table depicts coefficients (standard errors in parentheses) and *p*-values of the respective effects. Significant results in bold print. Number of observations = 460–1668, number of participants 235–242. TEAQ ASC = Touch Experiences and Attitudes Questionnaire (TEAQ) Attitude to self-care; TEAQ AUT = TEAQ Attitude to Unfamiliar Touch; TEAQ ChT = TEAQ Childhood Touch; TEAQ CIT = TEAQ Current Intimate Touch; TEAQ FFT = TEAQ Friends and Family Touch. ^a^ momentary affectionate touch levels (summed from self-reported types of touch); ^b^ 0 = male, 1 = female; ^c^ 0 = day1, 1 = day 2; ^d^ 0 = single, 1 = in a relationship; ^e^ time points over the day 1–6.

**Table 4:** Associations between different aspects of touch and hormonal states (cortisol and oxytocin) in everyday life.

Effects	Cortisol	Oxytocin
(A) Experiences and attitudes towards touch (TEAQ-G) as a predictor		
Fixed effects		
Intercept	1.863 (0.169); *p* < .001	4.717 (0.370); *p* < .001
TEAQ AIT	0.050 (0.041); *p* = .224	− 0.071 (0.089); *p* = .430
TEAQ ASC	0.029 (0.020); *p* = .148	0.075 (0.044); *p* = .092
TEAQ AUT	0.022 (0.022); *p* = .322	− 0.035 (0.049);*p* = .471
TEAQ ChT	0.004 (0.021); *p* = .851	− 0.023 (0.045); *p* = .611
TEAQ CIT	− 0.010 (0.035); *p* = .769	0.142 (0.074); *p* = .056
TEAQ FFT	− 0.052 (0.030); *p* = .085	0.018 (0.065); *p* = .787
Covariates		
Age	0.001 (0.002); *p* = .561	**− 0.013 (0.003); p < .001**
Sex^b^	0.040 (0.045); *p* = .366	− 0.126 (0.097); *p* = .198
Day^c^	− 0.013 (0.020); *p* = .519	− 0.011 (0.019); *p* = .588
Partner^d^	− 0.086 (0.055); *p* = .116	− 0.051 (0.019); *p* = .588
Body Mass Index	**− 0.012 (0.004); p = .002**	0.008 (0.008); *p* = .344
Random effects (SD)		
Intercept	0.198	0.173
Residual	0.086	0.110

(B) Attitude to intimate touch (TEAQ-G AIT) and momentary touch as predictors		
Fixed effects		
Within person		
Intercept	2.855 (0.128); *p* < .001	4.475 (0.250); *p* < .001
Touch^a^	0.014 (0.012); *p* = .231	− 0.022 (0.014); *p* = .104
Between person		
Touch^a^	− 0.030 (0.018); *p* = .099	0.005 (0.026); *p* = .848
TEAQ AIT	0.020 (0.035); *p* = .574	0.001 (0.072); *p* = .992
Touch^a^*TEAQ AIT	**− 0.078 (0.029); p = .007**	− 0.035 (0.043); *p* = .413
Covariates		
Age	− 0.001 (0.002); *p* = .456	**− 0.013 (0.003); p < .001**
Sex^b^	0.064 (0.044); *p* = .152	0.008 (0.093); *p* = .931
Day^c^	**− 0.041 (0.020); p = .044**	− 0.036 (0.024); *p* = .147
Partner^d^	− 0.066 (0.057); *p* = .242	0.119 (0.108); *p* = .274
Body Mass Index	**− 0.012 (0.004); p = .004**	0.007 (0.009); *p* = .404
Eating^e^	0.063 (0.047); *p* = .180	**0.164 (0.054); p = .003**
Physical activity^e^	− 0.026 (0.030); *p* = .390	0.032 (0.036); *p* = .368
Time(a)^f^	**0.078 (0.022); p < .001**	**− 0.199 (0.026); p < .001**
Time(b)^g^	**− 0.702 (0.021); p < .001**	**0.059 (0.024); p = .012**
Drinking^e^	0.005 (0.048); *p* = .912	0.020 (0.056); *p* = .724
Caffeine^e^	**0.080 (0.035); p = .024**	− 0.068 (0.041); *p* = .103
Cigarettes^e^	**0.182 (0.074); p = .014**	**0.191 (0.099); p = .054**
Random effects (SD)		
Intercept	0.058	0.001
Time^h^	0.040	0.033
Residual	0.379	0.453

(C) Childhood touch (TEAQ-G ChT) and momentary touch as predictors		
Fixed effects		
Intercept	2.825 (0.127); *p* < .001	4.458 (0.250); *p* < .001
Touch^a^	0.014 (0.012); *p* = .235	− 0.022 (0.014); *p* = .104
Between person		
Touch^a^	**− 0.036 (0.017); p = .039**	0.005 (0.025); *p* = .837
TEAQ ChT	− 0.023 (0.021); *p* = .290	− 0.008 (0.044); *p* = .863
Touch^a^*TEAQ ChT	**− 0.041 (0.018); p = .025**	− 0.034 (0.028); *p* = .235
Covariates		
Age	− 0.002 (0.002); *p* = .303	**− 0.012 (0.004); p < .001**
Sex^b^	0.075 (0.044); *p* = .089	0.012 (0.093); *p* = .901
Day^c^	− 0.039 (0.055); *p* = .474	− 0.036 (0.024); *p* = .138
Partner^d^	**− 0.042 (0.020); p = .040**	0.124 (0.106); *p* = .245
Body Mass Index	**− 0.011 (0.004); p = .007**	0.007 (0.009); *p* = .385
Eating^e^	0.060 (0.047); *p* = .200	**0.164 (0.054); p = .002**
Physical activity^e^	− 0.029 (0.030); *p* = .328	0.032 (0.036); *p* = .375
Time(a)^f^	**0.079 (0.022); p < .001**	**− 0.198 (0.026); p < .001**
Time(b)^g^	**− 0.703 (0.021); p < .001**	**0.059 (0.024); p = .013**
Drinking^e^	0.005 (0.049); *p* = .914	0.019 (0.056); *p* = .731
Caffeine^e^	**0.080 (0.035); p = .023**	− 0.068 (0.041); *p* = .102
Cigarettes^e^	**0.193 (0.073); p = .008**	**0.193 (0.099); p = .052**
Random effects (SD)		
Intercept	0.061	0.001
Time^h^	0.036	0.033
Residual	0.380	0.453

Table depicts unstandardized coefficients (standard errors in parentheses) and *p*-values of the respective effects. Significant results in bold print. Number of observations = 456 − 158. Number of participants = 227–234. Hormonal variables are log-transformed. TEAQ_ASC = Touch Experiences and Attitudes Questionnaire (TEAQ) Attitude to Selfcare; TEAQ AUT = TEAQ Attitude to Unfamiliar Touch; TEAQ ChT = TEAQ Childhood Touch; TEAQ CIT = TEAQ Current Intimate Touch; TEAQ FFT = TEAQ Friends and Family Touch. ^a^ momentary affectionate touch levels (summed from self-reported types of touch); ^b^ 0 = male, 1 = female; ^c^ 0 = day 1, 1 = day 2; ^d^ 0 = single, 1 = in a relationship; ^e^ 0 = no, 1 = yes; ^f^2 = time point 1, 1 = time point 2, 0 = time point 3–6 ; ^g^0 = time point 1–3, 1 = time point 4, 2 = time point 5, 3 = time point 6.; ^h^ time points over the day 1–6.

## Discussion

In this study, we assessed the validity of the German version of the Touch Experience and Attitude Questionnaire (TEAQ-G) and investigated how retrospectively reported childhood caregiver touch, as well as current attitudes towards and experiences of intimate touch, are associated with participants’ social relationships and mental health. Additionally, we examined how these subscales are related to individuals’ daily ratings of their emotional states and hormonal levels.

The factor analyses supported the expected six-factor structure, and internal consistency of the TEAG-G subscales was good to excellent, with Cronbach’s alpha ranging from α = 0.80 for the ASC to α = 0.93 for the CIT. These values were nearly identical to those in the original validation study^19^, supporting the reliability and structural validity of the TEAQ-G. Additionally, good convergent and criterion-related validity were demonstrated, as the TEAQ-G showed significant correlations with other validated measures, including the Social Touch Questionnaire (STQ)^24^, the Social Support Questionnaire (F-SozU)^26^, and the Childhood Trauma Questionnaire (CTQ)^25^. These findings align with our expectations and replicate results from the original validation paper: the STQ also correlated moderately to strongly with all TEAQ subscales except the TEAQ-G ASC^19^. Moreover, individuals who perceive higher social support are likely to have a larger social network, experience touch more frequently, and have a more positive attitude towards touch. Conversely, a positive experience with and attitude toward touch may contribute to developing a broader social network. Furthermore, as anticipated and reported before^42,43^, retrospectively reported touch experienced during childhood (TEAQ-G ChT) negatively correlated with CTQ scores, reflecting the expected association between childhood trauma and fewer positive touch experiences during early life. The explored associations between TEAQ-G and age revealed that the overall effect of age was moderate (partial η² =0.10), but the variance explained in individual subscales of TEAQ-G was small (0.4–2.3%) according to Cohen’s convention^44^.The results indicate that current intimate touch (TEAQ-G CIT) decreases with age, while touch with family and friends (TEAQ-G FFT) slightly increases. Additionally, older participants tend to report fewer childhood touch experiences (TEAQ-G ChT). This finding aligns with the previously reported low correlation in the Russian validation study of TEAQ, which suggested that older participants tended to receive slightly less affective touch in their childhood^45^. Interestingly, attitudes toward touch seem to be less influenced by age. While attitudes toward unfamiliar touch (TEAQ-G AUT) become slightly less positive with age, attitudes toward intimate touch (TEAQ-G AIT) and self-care (TEAQ-G ASC) remain relatively stable across different age groups. Overall, the TEAQ-G proves to be a valuable tool for the nuanced assessment of interpersonal touch. Unlike other existing touch questionnaires, which typically focus on either attitude toward touch or touch experiences, the TEAQ-G comprehensively captures both aspects while distinguishing between different touch contexts (familial, unfamiliar, and intimate). The detailed assessment can support research in clinical populations, such as patients with depression, trauma histories, or touch aversion. It can also help explore how individual differences (e.g., sensory sensitivity) and demographic factors (e.g., gender, cultural background) influence touch behavior. Moreover, combining TEAQ-G with neurophysiological and experimental studies, researchers can better link subjective touch experiences with biological (underlying) mechanisms. Finally, the TEAQ-G could contribute to intervention studies in clinical and caregiving contexts, providing insights into the role of positive touch across the life span.

The regression analyses examining the link between the TEAQ-G scores and social relationships and mental health aspects revealed several important associations. More positive retrospectively reported childhood touch experiences (TEAQ-G ChT) were associated with lower attachment avoidance, less family dysfunction, and lower levels of all measured outcomes of mental health impairment- including anxiety, depression, loneliness, and perceived stress - while being linked to higher resilience and general trust. Similarly, high levels of current intimate touch (TEAQ-G CIT) were linked to lower attachment anxiety and avoidance, better family functioning, greater relationship satisfaction, and improved mental health outcomes. These results align with previous research suggesting that early caregivers’ touch promotes a secure attachment style and reduces the likelihood of developing an avoidant attachment style in adulthood^1,3,4^. This positive impact of early tactile interaction throughout the lifespan is consistent with attachment theory, which conceptualizes affectionate touch as an expression of love and a sign of safety^46,47^. Affectionate parental touch provides children with warmth and protection, leading them to view other people as reliable and trustworthy, and has also been identified as a protective factor against depression^7^. Our findings not only support this research but also extend it, indicating that touch from loved ones in adulthood has an additional protective effect. Both retrospectively reported childhood touch and current intimate touch were associated with a reduced likelihood of depression, anxiety, and loneliness, as well as greater resilience. Consistent with these findings, prior research has shown that attachment anxiety is linked to a greater desire for and enjoyment of romantic partner touch, while attachment avoidance is associated with reduced touch engagement overall. Notably, individuals with high attachment anxiety benefit significantly from receiving touch, as it enhances their relational well-being. On the other hand, avoidantly attached individuals, despite their lower desire for touch, still experience similar positive effects from receiving affectionate touch^48^. Additionally, research by^49^ supports these findings, suggesting that avoidantly attached individuals engage in less touch, resulting in lower well-being. However, when avoidantly attached individuals do receive affectionate touch, they experience similar positive effects as less avoidantly attached individuals^49^.

Interestingly, a more positive attitude toward intimate touch (TEAQ-G AIT) was linked to higher attachment anxiety, lower attachment avoidance and family functioning, reduced relationship satisfaction, and greater feelings of loneliness. While this may seem counterintuitive at first, it is important to note that in this specific analysis, childhood touch and current intimate touch were controlled for and thus held constant in the model. So, this finding suggests that having a positive attitude toward touch can be problematic when accompanied by insufficient actual touch experiences. Supporting this, previous research has shown that longing for touch during periods of physical restrictions due to COVID-19 was associated with increased mental burden and lower quality of life^50,51^. Similarly, in our previous research, we found that individuals experiencing loneliness reported higher distress and anxiety when they had a more positive attitude toward social touch during the COVID-19-related lockdown^17^. Furthermore, the negative association found here may indicate that a more positive attitude toward intimate touch stems from less satisfying romantic and family relationships, making touch seem more desirable.

A similar pattern of results was observed in our ecological momentary assessment data using hierarchical linear models. First, we found that retrospectively reported childhood touch experiences (TEAQ-G ChT) was significantly associated with more favorable individuals’ daily emotional states, including lower stress, anxiety, loneliness, and pandemic-related burden, along with higher happiness. Second, current intimate touch experiences, as measured by TEAQ-G CIT, were also linked to lower stress, pandemic-related burden, and loneliness, as well as higher happiness and moderately higher oxytocin levels. Similarly, self-reported daily affectionate touch, measured during EMA, was significantly associated with lower pandemic-related burden, cortisol levels, and stress, while positively correlating with happiness. These findings align with existing research highlighting that pleasant touch is associated with decreased self-reported anxiety and stress levels^52^, as well as reduced cortisol^15,53^ and increased oxytocin levels both in laboratory settings^54^ and everyday life^17^. Moreover, these results fit well with the conclusions of^55^, who reviewed that affectionate touch consistently supports well-being across relational, psychological, and physical domains. However, it is important to note that in this study, the association between current touch and higher daily oxytocin levels was of marginal significance, so these results should be interpreted with caution. Nonetheless, they offer preliminary insights that could help guide future research on the nuanced relationship between affectionate touch and hormonal dynamics in everyday life. Moreover, we found that these positive associations are influenced by individuals’ childhood touch experiences (TEAQ-G ChT) and their attitude towards intimate touch (TEAQ-G AIT). More specifically, the associations between daily affectionate touch and lower stress, reduced Covid-19 burden, decreased cortisol levels, and higher happiness ratings was stronger among individuals with a more positive attitude toward intimate touch. These findings indicates that the positive effects of daily affectionate touch depend on how important and positive one’s attitude toward intimate touch is. This result is in line with previous EMA research showing associations between subjective levels of touch longing and pleasantness ratings of touch during the COVID-19 pandemic^56^. Interestingly, we found that childhood touch experiences enhanced the negative association between daily affectionate touch and both pandemic-related burden and cortisol levels, suggesting that these associations were more pronounced among individuals who reported having experienced greater levels of affectionate touch during childhood.This indicates that people who received little touch in childhood may benefit less from receiving more touch later in life, whereas those with more positive early touch experiences show greater benefits. These findings support the idea that early touch experiences can have long-lasting positive effects on individuals’ well-being, even at the hormonal level.

### Strengths and limitations

One of the strengths of this study is the large sample size, which enhances the statistical power of the validation analyses. We also aimed for age diversity and a balanced male-to-female ratio to improve the representativeness of our findings. According to the most recent data from the Federal Statistical Office of Germany (https://www.destatis.de/EN/Home/_node.html), our sample for TEAQ-G validation analyses is comparable to the general population. Although our sub-samples did not fully achieve representativeness regarding the female-to-male ratio and had a mean age skewing younger (approximately in the mid-thirties), we included a broad age range from 18 to 72. This variability helps us generalize our findings to a large proportion of the general population. However, since the questionnaire was validated in German, the sample shows limited cultural diversity. Additionally, we examined multiple aspects of touch in relation to social relationships, both baseline and daily mental health aspects, and hormonal levels in daily life. The use of ecological momentary assessment allowed us to gather ecologically valid data, capturing natural daily fluctuations in participants’ behaviors and emotions.

However, several limitations must be noted regarding this study. A part of the data was collected during the COVID-19 pandemic, which likely affected participants’ experiences of touch. Social interactions were restricted during this time, resulting in touch primarily occurring within family settings rather than with unfamiliar individuals. Additionally, the pandemic may have altered attitudes toward touch, leading to increased fear of infection or heightened sensitivity to touch in general. These unique circumstances limit the generalizability of the results to pre- or post-pandemic contexts. Additionally, pandemic-related time constraints prevented the thorough preregistration of hypotheses; therefore, statistical results should be interpreted with caution.

To mitigate the potential impacts of the pandemic and enhance our validation dataset, we collected additional data through Clickworker. This approach had both strengths and limitations. On the positive side, the sample characteristics were broadly preselected, yielding a diverse age range. Conversely, the less controlled nature of the data collection process may have resulted in careless or inattentive responses. However, we attempted to address this issue by implementing several attention checks and applying various criteria for data cleaning. We took particular care in preparing the data.

Lastly, it should be noted that early childhood touch was assessed retrospectively, which may be influenced by memory biases and individuals’ current psychological states. This limitation should be considered when interpreting the potential long-term effects of touch. Future research should aim to address this limitation by planning prospective longitudinal studies.

## Conlusion

Our study confirms the validity of the German version of the TEAQ and highlights the crucial role of affectionate touch in psychobiological well-being across the lifespan. Affectionate touch (measured by TEAQ-G or momentary self-reports) was associated with more functional social relationships, better mental health, including lower depression, anxiety, loneliness, and higher resilience. Additionally, affectionate touch was related to more positive daily emotional states, including lower stress, and pandemic-related burden, as well as hormonal changes, such as reduced cortisol and moderately elevated oxytocin levels. Importantly, the positive associations between daily affectionate touch and individuals’ emotional states and cortisol levels varied depending on early touch experiences and attitude toward intimate touch. Notably, individuals with a more positive attitude toward intimate touch benefited more from affectionate touch, while those who retrospectively reported more positive childhood touch experiences showed stronger associations between daily touch and reductions in cortisol and pandemic-related burden. These findings emphasize the long-lasting impact of early touch and its relevance for stress regulation and mental health in daily life.

## Supplementary Information

Below is the link to the electronic supplementary material.

Supplementary Material 1

Supplementary Material 2

Supplementary Material 3

Supplementary Material 4

Supplementary Material 5

## Data Availability

The datasets used and/or analysed during the current study available from the corresponding author on reasonable request.
