# Supplementary material for: Newly validated touch experiences and attitudes questionnaire in German (TEAQ-G) is linked to social functioning, mental health, and hormonal stress regulation
Source: Sci Rep. 2025 Oct 9;15:35228. doi: 10.1038/s41598-025-20885-y (PMC12511447; doi:10.1038/s41598-025-20885-y)
Supplement: Supplementary file 1 — Supplementary Material 1 [file 41598_2025_20885_MOESM1_ESM.pdf]

Touch Experiences and Attitudes Questionnaire (TEAQ) (reproduced with permission from Trotter et al., 2018)

Please select a response next to each of the statements below to indicate how much you agree or disagree with each statement

	Disagree strongly	Disagree a little	Neither agree nor disagree	Agree a little	Agree strongly
1. I dislike people being very physically affectionate towards me					
2. I like using body lotions					
3. I have to know someone quite well to enjoy a hug from them					
4. I find it natural to greet my friends and family with a kiss on the cheek					
5. There was a lot of physical affection during my childhood					
6. As a child I would often hug family members					
7. I like to use bath essence when having a bath					
8. I find stroking the hair of a person I am fond of very pleasurable					
9. My parents were not very physically affectionate towards me during my childhood					
10. I like to fall asleep in the arms of someone I am close to					
11. I often snuggle up on the sofa with someone					
12. I enjoy the physical intimacy of sexual foreplay					
13. I like to link arms with my friends and family as I walk along					
14. I usually hug my family and friends when I am saying goodbye					

15. As a child I found a hug from my parents when I was upset made me feel much happier					
16. It's nice when friends and family members greet me with a kiss					
17. I often hold hands with someone I know intimately					
18. When I am upset, there is usually someone who can comfort me.					
19. Kissing is a great way of expressing physical attraction					
20. It feels really good when someone I am fond of runs their fingers through my hair					
21. I regularly hug people I am close to					
22. As a child my parents would tuck me up in bed every night and give me a hug and a kiss goodnight to physically comfort me when I am upset					
23. My life lacks physical affection					
24. I enjoy having my skin stroked					
25. I often take a shower or bath with someone					
26. I enjoy having sex					
27. I often have sex					
28. I am put off by physical familiarity					
29. I can always find somebody					
30. I always greet my friends and family by giving them a hug					
31. I enjoy being cuddled by someone I am fond of					
32. My mother regularly bathed me as a child					
33. As a child my parents always comforted me when I was upset					

34. I enjoy the feeling of my skin against someone else's if I know them intimately					
35. As a child my parents would often hold my hand when I was walking along with them					
36. Most days I get a hug or a kiss					
37. If someone I don't know very well puts a friendly hand on my arm it makes me feel uncomfortable					
38. I often make physical contact with my friends and family when I am with them					
39. It makes me feel uncomfortable if someone I don't know very well touches me in a friendly manner					
40. I enjoy holding hands with someone I am fond of					
41. I often share a romantic kiss					
42. As a child my mother regularly brushed my hair					
43. I like exfoliating my skin					
44. Kissing is an enjoyable part of expressing romantic feeling					
45. I often have my skin stroked					
46. I often hold hands with someone I am fond of					
47. I like to stroke the skin of someone I know intimately					
48. I am on huggable terms with quite a few people					
49. I often fall asleep while holding someone I am close to					
50. Snuggling up on the sofa with someone is great					
51. I often put my arm around a close friend as we walk along together					
52. I like having a bath with lots of bubble bath					

53. I don’t get many hugs these days					
54. I am often given a shoulder massage					
55. I like to use face masks on my skin					
56. I like it when my friends and family greet me by giving me a hug					
57. I often link arms with my friends and family as I walk along					

Scoring: Disagree strongly = 1, disagree a little = 2, neither agree nor disagree = 3, agree a little = 4, agree strongly = 5

R denotes items which are reverse scored (i.e. disagree strongly = 5, disagree a little = 4, neither agree nor disagree = 3, agree a little = 2, agree strongly = 1). Item numbers below indicate the items which belong to each of the subscales

Calculate the mean score for each subscale to obtain a subscale score

Friends and family touch (FFT) (*11 items*): 4, 13, 14, 16, 21, 30, 38, 48, 51, 56, 57

Current intimate touch (CIT) (*14 items*): 11, 17, 18, 23**R**, 25, 27, 29, 36, 41, 45, 46, 49, 53**R**, 54

Childhood touch (*ChT*) (*9 items*): 5, 6, 9**R**, 15, 22, 32, 33, 35, 42

Attitude to self-care (*ASC*) (*5 items*): 2, 7, 43, 52, 55

Attitude to intimate touch (*AIT*) (*13 items*): 8, 10, 12, 19, 20, 24, 26, 31, 34, 40, 44, 47, 50

Attitude to unfamiliar touch (*AUT*) (*5 items*): 1**R,** 3**R**, 28**R**, 37**R**, 39**R**
