# Supplementary material for: Newly validated touch experiences and attitudes questionnaire in German (TEAQ-G) is linked to social functioning, mental health, and hormonal stress regulation
Source: Sci Rep. 2025 Oct 9;15:35228. doi: 10.1038/s41598-025-20885-y (PMC12511447; doi:10.1038/s41598-025-20885-y)
Supplement: Supplementary file 2 — Supplementary Material 2 [file 41598_2025_20885_MOESM2_ESM.pdf]

Touch Experiences and Attitudes Questionnaire - German (TEAQ-G)

Bitte wählen Sie neben jeder der folgenden Aussagen eine Antwort aus, um anzugeben, wie sehr Sie der jeweiligen Aussage zustimmen oder nicht zustimmen

	Stimme ganz und gar nicht zu	stimme eher nicht zu	weder noch	stimme etwas zu	stimme voll und ganz zu
1. Ich mag es nicht, wenn Menschen mir gegenüber körperlich sehr zugeneigt sind.					
2. Ich benutze gerne Körpercremes.					
3. Ich muss jemanden ziemlich gut kennen, um eine Umarmung von ihm/ihr zu genießen.					
4. Ich finde es natürlich, meine Freunde und Familie mit einem Kuss auf die Wange zu begrüßen.					
5. In meiner Kindheit habe ich viel körperliche Zuneigung erfahren.					
6. Als Kind habe ich oft meine Familienmitglieder umarmt.					
7. Ich verwende gerne Badezusätze, wenn ich ein Bad nehme.					
8. Ich finde es sehr angenehm, die Haare einer Person zu streicheln, die ich gernhabe.					
9. Meine Eltern sind während meiner Kindheit nicht sehr körperlich liebevoll mit mir umgegangen.					
10. Ich schlafe gerne in den Armen von jemandem ein, dem ich nahestehe.					
11. Ich kuschle oft mit jemandem auf dem Sofa.					
12. Ich genieße die körperliche Intimität während des sexuellen Vorspiels.					
13. Ich hake gerne die Arme bei meinen Freunden und meiner Familie ein, wenn wir zusammen gehen.					
14. Normalerweise umarme ich meine Familie und Freunde, wenn ich mich von ihnen verabschiede.					
15. Wenn ich als Kind traurig war/es mir als Kind nicht gut ging, fand ich, dass es mir durch eine Umarmung von meinen Eltern besser ging.					
16. Ich finde es schön, wenn Freunde und Familienmitglieder mich mit einem Kuss begrüßen.					
17. Ich halte oft Händchen mit jemandem, den ich innig kenne.					
18. Wenn ich traurig bin/es mir nicht gut geht, gibt es normalerweise jemanden, der mich trösten kann.					
19. Küssen ist eine tolle Art, körperliche Anziehung auszudrücken.					

20. Es fühlt sich wirklich gut an, wenn jemand, den ich gernhabe, mit den Fingern durch mein Haar fährt					
21. Ich umarme regelmäßig Menschen, denen ich nahe bin.					
22. Als Kind brachten mich meine Eltern jeden Abend ins Bett und gaben mir eine Umarmung und einen Gutenachtkuss.					
23. Meinem Leben fehlt es an körperlicher Zuneigung.					
24. Ich genieße es, wenn meine Haut gestreichelt wird.					
25. Ich gehe oft mit jemandem duschen oder baden.					
26. Ich genieße es, Sex zu haben.					
27. Ich habe oft Sex.					
28. In sozialen Situationen finde ich körperliche Berührung unangenehm.					
29. Ich kann immer jemanden finden, der mich mit körperlicher Zuneigung tröstet, wenn ich traurig bin/es mir nicht gut geht.					
30. Ich begrüße meine Freunde und Familie immer mit einer Umarmung.					
31. Ich genieße es, von jemandem, den ich gernhabe, geknuddelt zu werden.					
32. Meine Mutter hat mich als Kind regelmäßig gebadet.					
33. Als Kind haben mich meine Eltern immer getröstet, wenn ich traurig war/ es mir nicht gut ging.					
34. Ich mag das Gefühl, wenn meine Haut die Haut eines mir vertrauten Menschen berührt.					
35. Als Kind haben meine Eltern mich oft an der Hand gehalten, wenn ich mit ihnen gegangen bin.					
36. An den meisten Tagen bekomme ich eine Umarmung oder einen Kuss.					
37. Ich fühle mich unwohl, wenn jemand, den ich nicht sehr gut kenne, freundschaftlich die Hand auf meinen Arm legt.					
38. Ich suche oft Körperkontakt bei meinen Freunden und meiner Familie, wenn ich mit ihnen zusammen bin.					
39. Ich fühle mich unwohl, wenn mich jemand, den ich nicht sehr gut kenne, freundschaftlich berührt.					
40. Ich genieße es, Händchen mit jemandem zu halten, den ich gernhabe.					
41. Ich tausche oft romantische Küsse aus.					
42. Als Kind hat mir meine Mutter regelmäßig die Haare gebürstet.					

43. Ich mag es, ein Peeling für meine Haut zu benutzen.					
44. Küssen ist eine genussvolle Art, um romantische Gefühle auszudrücken.					
45. Ich lasse mir oft die Haut streicheln.					
46. Ich halte oft Händchen mit jemandem, den ich gernhabe.					
47. Ich streichle gerne die Haut von jemandem, den ich innig kenne.					
48. Es gibt schon ziemlich viele Menschen, mit denen ich knuddele.					
49. Ich schlafe oft ein, wenn ich jemanden im Arm halte, der mir nahe ist.					
50. Mit jemandem auf dem Sofa zu kuscheln ist großartig.					
51. Wenn ich mit einem engen Freund/einer engen Freundin zusammen gehe, lege ich oft meinen Arm um ihn/sie.					
52. Ich mag es, ein Bad mit viel Badeschaum zu nehmen.					
53. Ich werde zurzeit nicht oft umarmt.					
54. Ich bekomme oft eine Schultermassage.					
55. Ich benutze gerne Gesichtsmasken.					
56. Ich mag es, wenn meine Freunde und Familie mich mit einer Umarmung begrüßen.					
57. Ich hake oft die Arme mit meinen Freunden und meiner Familie ein, wenn wir zusammen gehen.					

Auswertung: Stimme ganz und gar nicht zu = 1, stimme eher nicht zu = 2, weder noch = 3, stimme etwas zu = 4, stimme voll und ganz zu = 5
R kennzeichnet invertierte Items (d. h. Stimme ganz und gar nicht zu = 5, stimme eher nicht zu = 4, weder noch = 3, stimme etwas zu = 2, stimme voll und ganz zu = 1).
Unten sind die Zuordnungen zu den jeweiligen Subskalen beschrieben.
Berechne den Mittelwert der Items jeder Subskala, um den Subskalenscore zu erhalten.

Berührungen durch Freunde und Familie (FFT) (11 Items): 4, 13, 14, 16, 21, 30, 38, 48, 51, 56, 57
Aktuelle intime Berührungen (CIT) (14 Items): 11, 17, 18, 23R, 25, 27, 29, 36, 41, 45, 46, 49, 53R, 54
Berührungen in der Kindheit (ChT) (9 Items): 5, 6, 9R, 15, 22, 32, 33, 35, 42
Einstellung zur Selbstfürsorge (ASC) (5 Items): 2, 7, 43, 52, 55
Einstellung zu intimen Berührungen (AIT) (13 Items): 8, 10, 12, 19, 20, 24, 26, 31, 34, 40, 44, 47, 50
Einstellung zu Berührungen mit wenig vertrauten Menschen (AUT) (5 Items): 1R, 3R, 28R, 37R, 39R
