# Supplementary material for: Newly validated touch experiences and attitudes questionnaire in German (TEAQ-G) is linked to social functioning, mental health, and hormonal stress regulation
Source: Sci Rep. 2025 Oct 9;15:35228. doi: 10.1038/s41598-025-20885-y (PMC12511447; doi:10.1038/s41598-025-20885-y)
Supplement: Supplementary file 3 — Supplementary Material 3 [file 41598_2025_20885_MOESM3_ESM.docx]

**Appendix 3: Results of regression analyses with TEAQ-G predicting outcomes of social relationships and mental health aspects**

| Outcome | | Social Relationships | | | | |  | Mental Health Aspects | | | |
| --- | --- | --- | --- | --- | --- | --- | --- | --- | --- | --- | --- |
|  |  | ECR anxiety | ECR avoidance | PFB | Score-15 | GTS |  | HADS | UCLA Loneliness | PSS | BRS |
| Constant | b  (SE; p) | 16.25  (1.82; <.001) | 22.32  (1.45; <.001) | 16.61  (1.86; <.001) | 3.51  (.21; <.001) | 2.59  (.20; <.001) |  | 27.67  (2.34; <.001) | 63.42  (3.19; <.001) | 41.61  (2.50; <.001) | 2.29  (.26; <.001) |
| FFT | β  (SE; p) | **.16**  **(.39; .004)** | .08  (.31; .140) | -.07  (.37; .258) | .04  (.05; .404) | .12  (.04; .035) |  | .13  (.51; .021) | -.05  (.69; .363) | .12  (.55; .031) | **-.17**  **(.06; .003)** |
| CIT | β  (SE; p) | **-.52**  **(.44; <.001)** | **-.34**  **(.35; <.001)** | **.68**  **(.43; <.001)** | **-.34**  **(.05; <.001)** | .08  (.05; .251) |  | **-.47**  **(.58; <.001)** | **-.60**  **(.79; <.001)** | **-.42**  **(.62; <.001)** | **.34**  **(.06; <.001)** |
| ChT | β  (SE; p) | -.05  (.28; .256) | **-.13**  **(.22; .003)** | -.01  (.28; .847) | **-.37**  **(.03; <.001)** | **.13**  **(.03; .008)** |  | **-.21**  **(.36; <.001)** | **-.19**  **(.49; <.001)** | **-.18**  **(.39; .001)** | **.16**  **(.04; .001)** |
| ASC | β  (SE; p) | .03  (.25; .475) | **.12**  **(.20; .005)** | -.04  (.23; .364) | **.12**  **(.03; .004)** | -.08  (.03; .092) |  | **.11**  **(.33; .011)** | .06  (.45; .119) | .10  (.35; .031) | **-.13**  **(.04; .007)** |
| AIT | β  (SE; p) | **.22**  **(.49; .002)** | **-.24**  **(.39; <.001)** | **-.21**  **(.49; .001)** | **.18**  **(.06; .002)** | -.04  (.05; .499) |  | .10  (.64; .084) | **.22**  **(.88; <.001)** | .14  (.69; .015) | -.09  (.07; .158) |
| AUT | β  (SE; p) | **-.13**  **(.31; .009)** | -.01  (.25; .840) | -.06  (.28; .221) | **-.13**  **(.04; .006)** | **.15**  **(.03; .003)** |  | **-.18**  **(.41; <.001)** | **-.14**  **(.55; .001)** | **-.21**  **(.43; <.001)** | **.19**  **(.05; .001)** |
| Sex ^a^ | β  (SE; p) | -.03  (.29; .430) | -.02  (.23; .672) | -.06  (.23; .166) | -.06  (.03; .111) | -.03  (.03; .482) |  | .02  (.34; .578) | .02  (.46; .676) | .06  (.36; .146) | -.01  (.04; .747) |
| Partner ^b^ | β  (SE; p) | -.00  (.71; .966) | -.06  (.56; .235) | – | .05  (.08; .352) | -.01  (.08; .801) |  | .16  (.93; .003) | .07  (1.26; .152) | **.19**  **(.99; .001)** | -.10  (.10; .086) |
| Age | β  (SE; *p*) | **-.23**  **(.02; <.001)** | -.03  (.02; .444) | **-.17**  **(.02; .001)** | **-.32**  **(.00; <.001)** | .09  (.00; .063) |  | **-.24**  **(.03; <.001)** | **-.14**  **(.03; .001)** | **-.29**  **(.03; <.001)** | **.22**  **(.00; <.001)** |
| **Model** | **R^2^**  **(F; p)** | **.19**  **(12.203; <.001)** | **.31**  **(23.288; <.001)** | **.38**  **(25.853; <.001)** | **.26**  **(19.044; <.001)** | **.10**  **(6.189; <.001)** |  | **.20**  **(13.202; <.001)** | **.33**  **(26.780; <.001)** | **.17**  **(11.212; <.001)** | **.13**  **(7.715; <.001)** |

*Note. N* = 344–527. Standardized coefficients (β), standard errors (SE), and p-values are displayed. Bold values indicate p-values significant at Bonferroni-adjusted α. Abbr. outcomes. FFT = Touch Experiences and Attitudes Questionnaire (TEAQ) Friends and Family Touch; CIT = TEAQ Current Intimate Touch; ChT = TEAQ Childhood Touch; ASC = TEAQ Attitude to self-care; AIT = TEAQ Attitude to Intimate Touch; AUT = TEAQ Attitude to Unfamiliar Touch; ECR = Experience in Close Relationships Scale; PFB = Partnership Questionnaire; Score-15 = Systemic Clinical Outcome and Routine Evaluation 15 (family functioning); GTS = General Trust Scale; HADS = Hospital Anxiety and Depression Scale; UCLA Loneliness = Loneliness Scale; PSS = Perceived Stress Scale; BRS = Brief Resilience Scale. ^a^ 0 = male, 1 = female. ^b^ 0 = no, 1 = yes.
