# Supplementary material for: Newly validated touch experiences and attitudes questionnaire in German (TEAQ-G) is linked to social functioning, mental health, and hormonal stress regulation
Source: Sci Rep. 2025 Oct 9;15:35228. doi: 10.1038/s41598-025-20885-y (PMC12511447; doi:10.1038/s41598-025-20885-y)
Supplement: Supplementary file 4 — Supplementary Material 4 [file 41598_2025_20885_MOESM4_ESM.docx]

| **Appendix 2:** *Sociodemographic Characteristics of Participants* | | | | | | | | | | | | |
| --- | --- | --- | --- | --- | --- | --- | --- | --- | --- | --- | --- | --- |
|  | Sample used for validation analyses (N=1319) | | | | Sample used for regression analyses (N=629) | | | | Sample used for hierarchical linear models (N=253) | | | |
|  | *M* | *SD* | *Min* | *Max* | *M* | *SD* | *Min* | *Max* | *M* | *SD* | *Min* | *Max* |
| Age | 37.41 | 13.96 | 18 | 81 | 34.80 | 14.50 | 18 | 81 | 34 | 13.18 | 19 | 79 |
|  | *n* | | *%* | | *n* | | *%* | | *n* | | *%* | |
| Sex |  | |  | |  | |  | |  | |  | |
| Male | 660 | | *50* | | 129 | | *20.5* | | 74 | | *29.2* | |
| Female | 644 | | *48.8* | | 494 | | *78.5* | | 178 | | *70.4* | |
| Other | 10 | | *0.8* | | 3 | | *0.5* | | - | | *-* | |
| Prefer not to answer | 5 | | *0.4* | | 3 | | *0.5* | | 1 | | *0.4* | |
| Sexual Orientation |  | |  | |  | |  | |  | |  | |
| Heterosexual | 1120 | | *84.9* | | 533 | | *84.7* | | 217 | | *85.8* | |
| Homosexual | 44 | | *3.3* | | 19 | | *3.0* | | 8 | | *3.2* | |
| Bisexual | 77 | | *5.8* | | 49 | | *7.8* | | 22 | | *8.7* | |
| Other | 50 | | *3.8* | | 12 | | *1.9* | | 3 | | *1.2* | |
| Prefer not to answer | 10 | | *0.8* | | 16 | | *2.5* | | 3 | | *1.2* | |
| Missing | 18 | | *1.4* | | - | | *-* | | - | | *-* | |
| Country of Living |  | |  | |  | |  | |  | |  | |
| Germany | 1218 | | *92.3* | | 614 | | *97.6* | | 252 | | *99.6* | |
| Austria | 65 | | *4.9* | | 7 | | *1.1* | | - | | *-* | |
| Switzerland | 14 | | *1.1* | | 3 | | *0.5* | | - | | *-* | |
| Other | 7 | | *0.5* | | 5 | | *0.8* | | - | | *-* | |
| Missing | 15 | | *1.1* | | - | | *-* | | 1 | | *0.4* | |
| Size of residential area |  | |  | |  | |  | |  | |  | |
| < 10.000 inhabitants | 314 | | *23.8* | | 116 | | *18.4* | | 42 | | *16.6* | |
| ≤ 100.000 inhabitants | 364 | | *27.6* | | 164 | | *26.1* | | 59 | | *23.3* | |
| > 100.000 inhabitants | 443 | | *33.6* | | 298 | | *47.4* | | 132 | | *52.2* | |
| > 1.000.000 inhabitants | 176 | | *13.3* | | 47 | | *7.5* | | 20 | | *7.9* | |
| Prefer not to answer | 7 | | *0.5* | | 4 | | *0.6* | | - | | *-* | |
| Missing | 15 | | *1.1* | | - | | *-* | | - | | *-* | |
| Formal Education |  | |  | |  | |  | |  | |  | |
| Finished school without diploma | 2 | | *0.2* | | - | | *-* | | - | | *-* | |
| Secondary school diploma/ leaving certificate | 82 | | *6.2* | | 25 | | *4* | | 9 | | *3.6* | |
| Finished Apprenticeship | 185 | | *14* | | 46 | | *7.3* | | 20 | | *7.9* | |
| University entrance qualification | 377 | | *28.6* | | 199 | | *31.6* | | 83 | | *32.8* | |
| University Degree | 610 | | *46.2* | | 307 | | *48.8* | | 121 | | *47.8* | |
| Missing | 63 | | *4.8* | | 52 | | *8.3* | | 20 | | *7.9* | |
| Employment ^a^ |  | |  | |  | |  | |  | |  | |
| Pupil/Apprentice/University Student | 150 | | *11.4* | | 125 | | *19.9* | | 55 | | *21.7* | |
| Employee/public official | 675 | | *51.2* | | 283 | | *45.0* | | 106 | | *41.9* | |
| Self-employed | 172 | | *13.0* | | 37 | | *5.9* | | 11 | | *4.3* | |
| Unemployed/Job Seeking | 64 | | *4.9* | | 17 | | *2.7* | | 8 | | *3.2* | |
| Retired/homemaker | 122 | | *9.2* | | 53 | | *8.4* | | 21 | | *8.3* | |
| Income |  | |  | |  | |  | |  | |  | |
| No Personal income | 116 | | *8.8* | | 99 | | *15.7* | | 38 | | *15.0* | |
| ≤ 2000 | 602 | | *46.0* | | 294 | | *46.7* | | 129 | | *51.0* | |
| ≤ 4000 | 394 | | *29.9* | | 171 | | *27.2* | | 71 | | *28.1* | |
| > 4000 | 77 | | *5.8* | | 33 | | *5.2* | | 11 | | *4.3* | |
| Prefer not to/can’t answer | 110 | | *8.3* | | 32 | | *5.1* | | 4 | | *1.6* | |
| Missing | 15 | | *1.1* | | - | | *-* | | - | | *-* | |
| Relationship Status ^a^ |  | |  | |  | |  | |  | |  | |
| Married and living together | 390 | | *29.6* | | 155 | | *24.6* | | 53 | | *20.9* | |
| In a relationship (living together) | 244 | | *18.5* | | 138 | | *21.9* | | 59 | | *23.3* | |
| In a relationship (living separately) | 175 | | *13.3* | | 110 | | *17.5* | | 61 | | *24.1* | |
| In a partner-like relationship | 49 | | *3.7* | | 31 | | *4.9* | | 14 | | *5.5* | |
| Single | 431 | | *32.7* | | 177 | | *28.1* | | 57 | | *22.5* | |
| Divorced or similar | 60 | | *4.5* | | 35 | | *5.6* | | 14 | | *5.5* | |
| Widowed | 10 | | *0.8* | | 5 | | *0.8* | | 2 | | *0.8* | |
| Living situation |  | |  | |  | |  | |  | |  | |
| Living alone | 342 | | *25.9* | | 137 | | *21.8* | | 51 | | *20.2* | |
| Living with others | 962 | | *72.9* | | 429 | | *78.2* | | 202 | | *79.8* | |
| Missing | 15 | | *1.1* | | - | | *-* | | - | | *-* | |
| Children |  | |  | |  | |  | |  | |  | |
| Yes | 419 | | *67.2* | | 193 | | *30.7* | | 73 | | *28.9* | |
| No | 887 | | *31.8* | | 436 | | *69.3* | | 180 | | *71.1* | |
| Missing | 13 | | *1.0* | | - | | *-* | | - | | *-* | |

*Note.* ^a^ For this question, multiple answers were allowed, so participants could select more than one option.
