# Supplementary figures and images for: Newly validated touch experiences and attitudes questionnaire in German (TEAQ-G) is linked to social functioning, mental health, and hormonal stress regulation
Source: Sci Rep. 2025 Oct 9;15:35228. doi: 10.1038/s41598-025-20885-y (PMC12511447; doi:10.1038/s41598-025-20885-y)

## Appendix 1c\_CFA

Figure 1: CFA Original Model

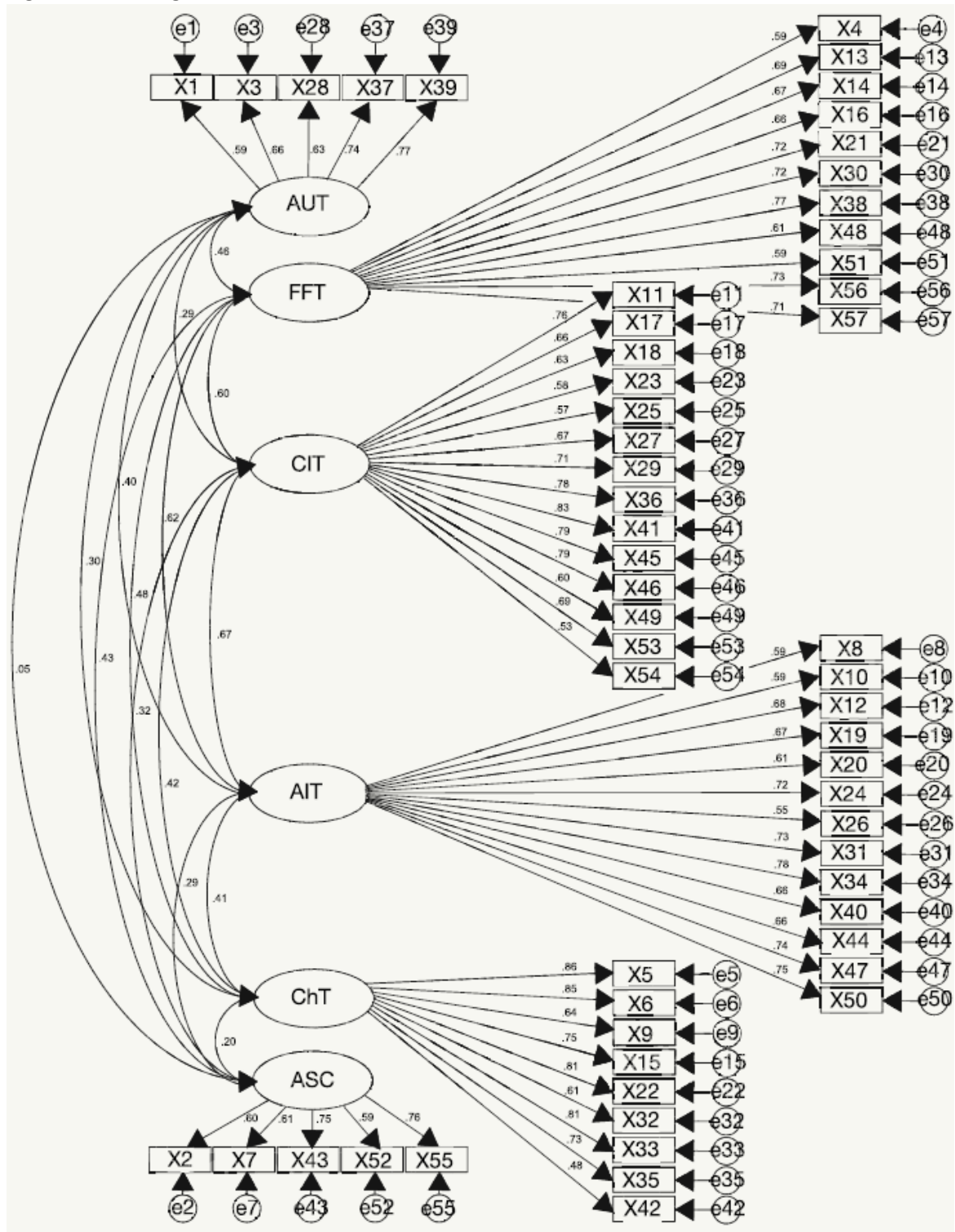

Figure 2: CFA Parceled Model

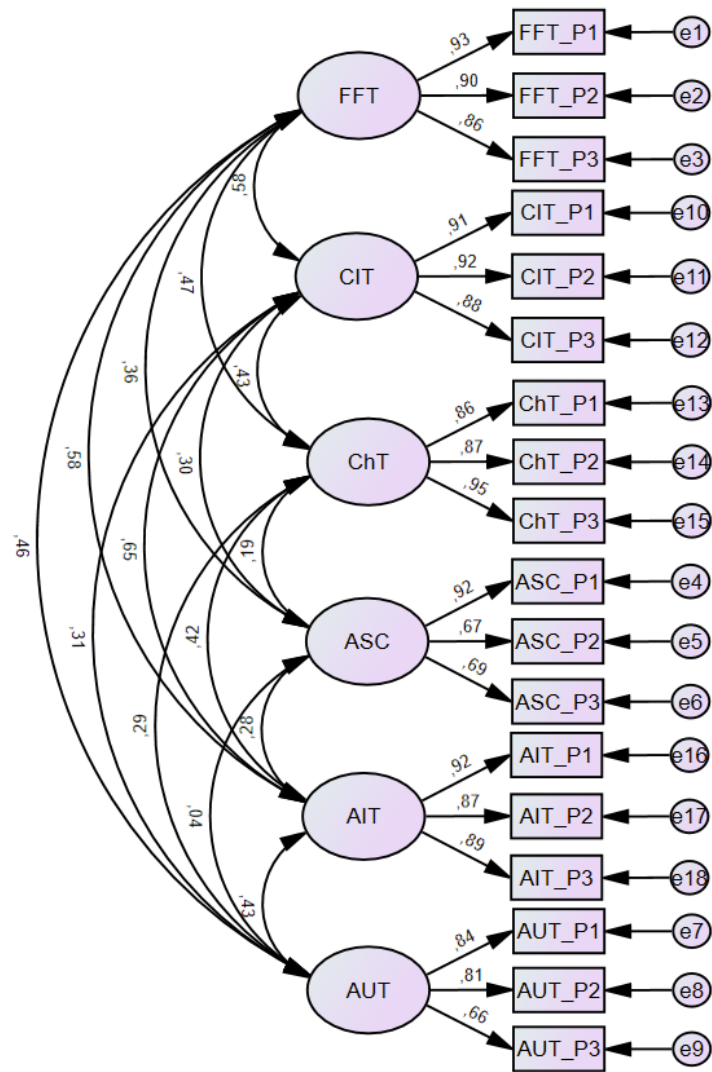

Supplement: Supplementary file 5 — Supplementary Material 5 [file 41598_2025_20885_MOESM5_ESM.pdf]
